# Efficacy and safety of transcatheter arterial embolization for lower gastrointestinal bleeding: a systematic review and meta-analysis of 58 clinical trials

**DOI:** 10.1186/s40001-025-03605-0

**Published:** 2025-12-02

**Authors:** Qiulian Sun, Jiefei Cheng, XueLei Zhang, Xiangzhong Huang, Ling Tang, Jingjing Li, Dongqing Ren, Xinjian Xu, Delei Cheng

**Affiliations:** 1 Department of Radiology, The Fifth People’s Hospital of Suzhou, The Affiliated Infectious Diseases Hospital, Suzhou Medical College of Soochow University, No.10, Guangqian Road, Suzhou, 215100 Jiangsu Province China; 2 Department of Radiology, The Fifth People’s Hospital of Taizhou, No.51, Yunhe Road, Tai Zhou City, 225300 Jiangsu Province China; 3 Department of Emergency, Jiangyin Hospital Affiliated to Nantong University, No. 3, Yingrui Road, Jiang Yin City, 214400 China; 4 Department of Interventional Radiology, Jiangyin Hospital Affiliated to Nantong University, No. 3, Yingrui Road, Jiang Yin City, 214400 China; 5 Department of Radiology, Taizhou Fourth People’s Hospital, No.99, Gulou Road, Tai Zhou City, 225300 Jiangsu Province China; 6 Department of Radiology, Jiangyin Traditional Chinese Medicine Orthopedic Hospital, No. 41, Middle Street, Yunting Town, Jiang Yin City, 214400 Jiangsu Province China; 7 Department of Interventional Radiology, The First Affiliated Hospital of USTC, Division of Life Sciences and Medicine, University of Science and Technology of China, No.17 Lujiang Road, Hefei City, 230000 China; 8 Yangtze University, Jingzhou City, 434020 Hubei Province China

**Keywords:** Acute lower gastrointestinal bleeding, Transcatheter arterial embolization, Meta-analysis

## Abstract

**Objective:**

Acute lower gastrointestinal bleeding (LGIB) is a common and life-threatening condition, particularly in elderly patients, characterized by high morbidity and mortality rates. While transcatheter arterial embolization (TAE) has been widely adopted as an essential interventional treatment, its efficacy and safety have yet to be thoroughly and systematically evaluated. This study aims to assess the safety, efficacy, and clinical outcomes of TAE for acute LGIB through a meta-analysis, thereby providing valuable evidence to inform clinical decision-making.

**Methods:**

A comprehensive search was conducted across PubMed, Embase, Web of Science, and Cochrane databases. Studies meeting predefined criteria were selected, and key clinical outcomes, including the technical success rate, clinical success rate, rebleeding rate, and complication rate of TAE in LGIB treatment, were extracted. A random-effects model was employed for data integration, and heterogeneity was assessed using the *I*^2^ statistic. The quality of the included studies was evaluated, and publication bias was examined using funnel plots and Egger's test. Subgroup analyses were performed based on the use of TAE as a first-line treatment, the type of embolic materials used, and the year of publication.

**Results:**

A total of 58 studies involving 2262 patients were included. The technical success rate of TAE was 97.7% (95% CI 96.4–98.9%); the clinical success rate was 75.0% (95% CI 71.3–78.6%); the rebleeding rate was 17.3% (95% CI 14.7–20.1%); the rate of major complications was 4.3% (95% CI 3.0–5.8%); the rate of mild ischemia was 4.7% (95% CI 2.9–6.7%); and the rate of secondary complications was 10.8% (95% CI 7.5–14.6%). Subgroup analysis revealed that, since 2010, both the efficacy and safety of TAE have improved, with a notable reduction in overall ischemic events (including both severe and mild intestinal ischemia) from 12.7% to 6.3%.

**Conclusion:**

TAE is an effective and safe interventional treatment for acute LGIB, demonstrating high technical and clinical success rates. However, the occurrence of ischemia and complications continues to present significant challenges. Future advancements in technology are expected to further improve treatment outcomes.

**Supplementary Information:**

The online version contains supplementary material available at 10.1186/s40001-025-03605-0.

## Introduction

Acute lower gastrointestinal bleeding (LGIB) refers to bleeding originating from the distal aspect of the Treitz ligament, commonly presenting as hematochezia or melena [1, 2]. As the global population ages, the incidence of LGIB has risen steadily, particularly among individuals over 80 years old, where both the incidence and mortality rates have shown a notable increase. Research indicates that the annual incidence of LGIB in those under 60 years old is 92 per 100,000, compared to 877 per 100,000 in individuals aged 60 and above, with mortality rates ranging between 3.4% and 8.8% [3, 4]. This trend not only reflects shifts in population demographics but also underscores the substantial role of factors such as multiple comorbidities and the use of anticoagulant medications in elevating the risk of bleeding. As a result, the effective management of acute and complex LGIB cases has become a significant challenge in contemporary clinical practice.

Although conservative management is effective for most patients with LGIB, around 10–15% of cases require additional intervention [5]. Endoscopic therapy is widely recommended as the first-line treatment in LGIB management guidelines (ACG, ESGE) [1, 6]. However, for patients experiencing hemodynamic instability, those unable to undergo endoscopy, or those with persistent bleeding despite failed endoscopic attempts, transcatheter arterial embolization (TAE) has emerged as an essential intervention. As a key vascular therapeutic procedure, TAE has become increasingly crucial in managing complex LGIB cases [7]. Several clinical guidelines [1, 6, 8], including ACG, ESGE, and CSDE, recommend prioritizing TAE for patients who are ineligible for endoscopy or who have not responded to endoscopic treatment (guideline recommendation level: strong, evidence quality level: low).

Although TAE has been utilized for decades in the management of LGIB, studies assessing its rebleeding and complication rates demonstrate significant variability. The reported early rebleeding rates after TAE range from 3.8% to 28.6% [9, 10], while the incidence of intestinal ischemia and necrosis varies between 0% and 26.7% [11, 12], and the occurrence of secondary complications such as nephrotoxicity, hematoma, pain, and fever ranges from 4.5% to 41% [13, 14]. These wide fluctuations underscore the heterogeneity in factors such as patient characteristics, treatment protocols, and embolic materials across different studies, contributing to a lack of standardized treatment guidelines and clearly defined safety thresholds. Moreover, compared to research on upper gastrointestinal bleeding (UGIB), the evidence supporting the management of LGIB remains relatively sparse. The field of LGIB is particularly lacking in high-quality randomized controlled trials (RCTs), with most data still derived from observational studies. As a result, the establishment of optimal treatment practices and the evaluation of efficacy for LGIB are still in an early stage of exploration.

Recent advances in imaging technology, particularly the use of computed tomography angiography (CTA), have significantly improved both the diagnosis and management of acute LGIB. As a high-sensitivity, non-invasive imaging modality, CTA not only allows for accurate localization of the bleeding source but also enables real-time monitoring of blood flow, detection of extravasation, and precise guidance for TAE. Both the latest BSG guidelines [15] and the updated ACG guidelines [16] now recommend the combined use of CTA and TAE as the standard approach, particularly when CTA reveals signs of extravasation.

Despite the progress made, the treatment of LGIB continues to present significant challenges, primarily due to the absence of high-quality clinical trial data. The heterogeneity and limitations inherent in existing studies contribute to uncertainties regarding the assessment of efficacy and safety. To address this gap, the present study aims to systematically evaluate the safety and efficacy of TAE in LGIB treatment through a meta-analysis of large-scale observational data. By synthesizing real-world evidence from existing studies, this meta-analysis seeks to provide more accurate and reliable data to inform clinical decision-making. In the absence of large-scale, multicenter randomized controlled trials, the findings of this study hold considerable practical and clinical value.

## Materials and methods

### Methods and search strategy

This meta-analysis was conducted and reported in strict accordance with the PRISMA guidelines [17]. Although the study protocol was not prospectively registered on an international platform such as PROSPERO, all study procedures, including the literature search, screening criteria, data extraction, and planned analyses, were defined a priori before the initiation of the systematic review. A comprehensive systematic literature search was performed in PubMed, Embase, Web of Science, and the Cochrane Library databases from their inception to [Last Search Date] without language restrictions. The search strategy combined controlled vocabulary (e.g., MeSH terms including “Lower Gastrointestinal tract,” “Hemorrhage,” “Gastrointestinal Hemorrhage,” “Angiography,” and “Radiography, Interventional”) with free-text terms (e.g., “lower gastrointestin,” “lower GI,” “colonic,” “catheter angiograph,” “transarterial”). Search terms were combined using Boolean operators (AND, OR) across the three conceptual domains of lower gastrointestinal, hemorrhage, and intervention. Full search strategies are available in Supplementary Materials-1. In addition, a manual search was conducted by reviewing the reference lists of studies included in prior systematic reviews and pertinent articles. The search covered literature from the inception of the databases up to November 1, 2024.

### Inclusion and exclusion criteria

Inclusion Criteria: (1) Articles written in English. (2) RCTs, retrospective cohort studies, prospective cohort studies, and case series with a sample size greater than 10. (3) Studies employing TAE for LGIB were included. LGIB was defined as hemorrhage originating distal to the ligament of Treitz, including bleeding from both the small intestine and colorectum. (4) Studies reporting at least one of the following outcome measures: technical success rate, clinical success rate, rebleeding rate, ischemia rate, or complication rate. Exclusion Criteria: (1) Duplicate publications. (2) Full text not accessible. (3) Grey literature and other specific publication types (e.g., case reports, reviews, comments, conference abstracts, dissertations, ongoing trials). (4) Studies with incomplete or missing essential data.

### Data extraction and definition

Data extraction was independently performed by two investigators, and any discrepancies were resolved through consultation and consensus. The following information was extracted from the included studies: authors, year of publication, study design, location (country or region), number of patients, embolization materials used, technical success rate, clinical success rate, secondary surgery rate, re-bleeding intervention rate, major complications, secondary complications, and first-line treatment.

The technical success rate was defined as the successful implementation of the embolization technique. The clinical success rate was defined as no need for further intervention (endoscopy or surgery) due to bleeding within 30 days after the initial intervention, and no major complications or death within 30 days of the procedure. The secondary surgery rate was defined as the need for surgery due to tumor or other reasons after the initial embolization. The re-bleeding intervention rate was defined as the need for re-intervention (endoscopic or surgical) due to re-bleeding within 30 days.

Ischemic complications were classified according to the Society of Interventional Radiology (SIR) reporting standards [18]. Major complications included any unplanned increase in the level of care, prolonged hospitalization, permanent adverse sequelae, or death, with severe ischemia-related complications such as intestinal infarction, muscle fibrosis, or intestinal obstruction being mainly reported. Secondary complications resulted in no long-term sequelae and required minimal treatment, including mild ischemic complications (e.g., mucosal ulcers or ischemia), fever, pain, and other complications related to interventional procedures.

### Quality assessment

The risk of bias for the included studies was assessed using appropriate, internationally recognized tools tailored to each study design. Specifically, the JBI [19] critical appraisal checklist was employed for single-arm case series studies, and the Newcastle–Ottawa Scale [20] was used for observational cohort studies. All assessments were conducted independently by two reviewers, with any discrepancies resolved through consensus.

### Statistical analysis

All statistical analyses were performed using Stata 11.0. Pooled estimates with 95% confidence intervals (CIs) were calculated using a random-effects model. The Freeman-Tukey double arcsine transformation was applied to stabilize variances before pooling proportions. Results are presented in forest plots. Heterogeneity was explored via subgroup and sensitivity analyses, while publication bias was assessed using funnel plots and Egger's test, with a significance threshold of *P* < 0.05.

## Result

### Retrieval results of literature

**Fig. 1:**
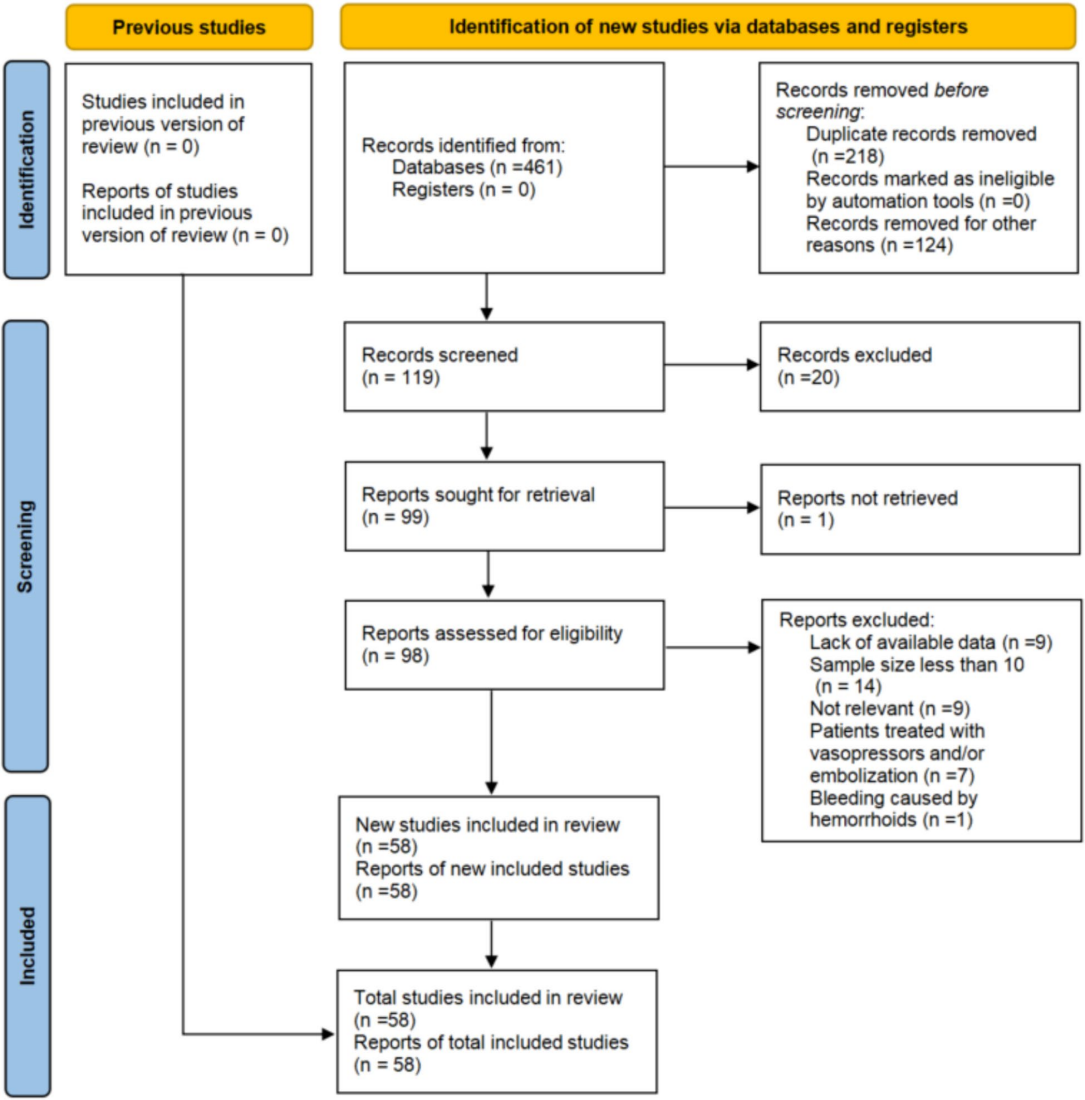
PRISMA 2020 flow diagram

The initial literature search identified 461 records. After applying the inclusion and exclusion criteria, 58 studies involving a total of 2262 patients were included for analysis. The study selection process is detailed in the PRISMA flow diagram (Fig. 1). The general characteristics of the included studies are summarized in Table 1, with more detailed data provided in Supplementary Table 1.

**Table 1:** Summary characteristics of the included studies (*n* = 58)

Characteristics	Categories	Number of studies (*n*)	Percentage (%)	Remarks
Research design	Retrospective study	58	100%	/
Publication year	1980–1989	1	1.7%	/
	1990–1999	4	6.9%	/
	2000–2009	18	31.0%	/
	2010–2019	26	44.8%	Peak of research
	2020–2024	9	15.5%	/
Region	North America	19	32.8%	Including the United States, Canada, etc
	Europe	9	15.5%	Including the United Kingdom, Switzerland, etc
	Asia	21	36.2%	Including China, South Korea, Japan, etc
	Others	9	15.5%	Including Australia, New Zealand, Brazil, etc
Embolic materials	Coil alone	8	13.8%	/
	NBCA alone	3	5.2%	/
	Gelfoam alone	1	1.7%	/
	Combination	41	70.7%	Most studies employed a combination of embolic materials
	Not reported	5	8.6%	/
Number of included studies		58	100%	
Sample size (patients)		/	/	Average: 39, Range: 10 – 134

### Quality assessment of the included studies

The quality of the 56 included retrospective case series was assessed using the JBI Critical Appraisal Checklist for Case Series. Overall, the studies demonstrated a low risk of bias. Specifically, the proportions of low-risk ratings were 94.6% (53/56) for item Q8 and 96.4% (54/56) for Q9. In contrast, 23.2% (13/56) of studies were rated as at risk for item Q4. For all remaining assessment items (Q1–Q3, Q5–Q7, Q10), the results indicated a low risk of bias across all included studies (100%). The two observational cohort studies were evaluated using the Newcastle–Ottawa Scale, both indicating high quality (scores of 9 and 8, respectively). Detailed quality assessment results are provided in Supplementary Table 2, with summary bar charts of the quality domains presented in Supplementary Figs. 1 and 2.

### Meta-analysis results

#### Technical success rate

**Fig. 2:**
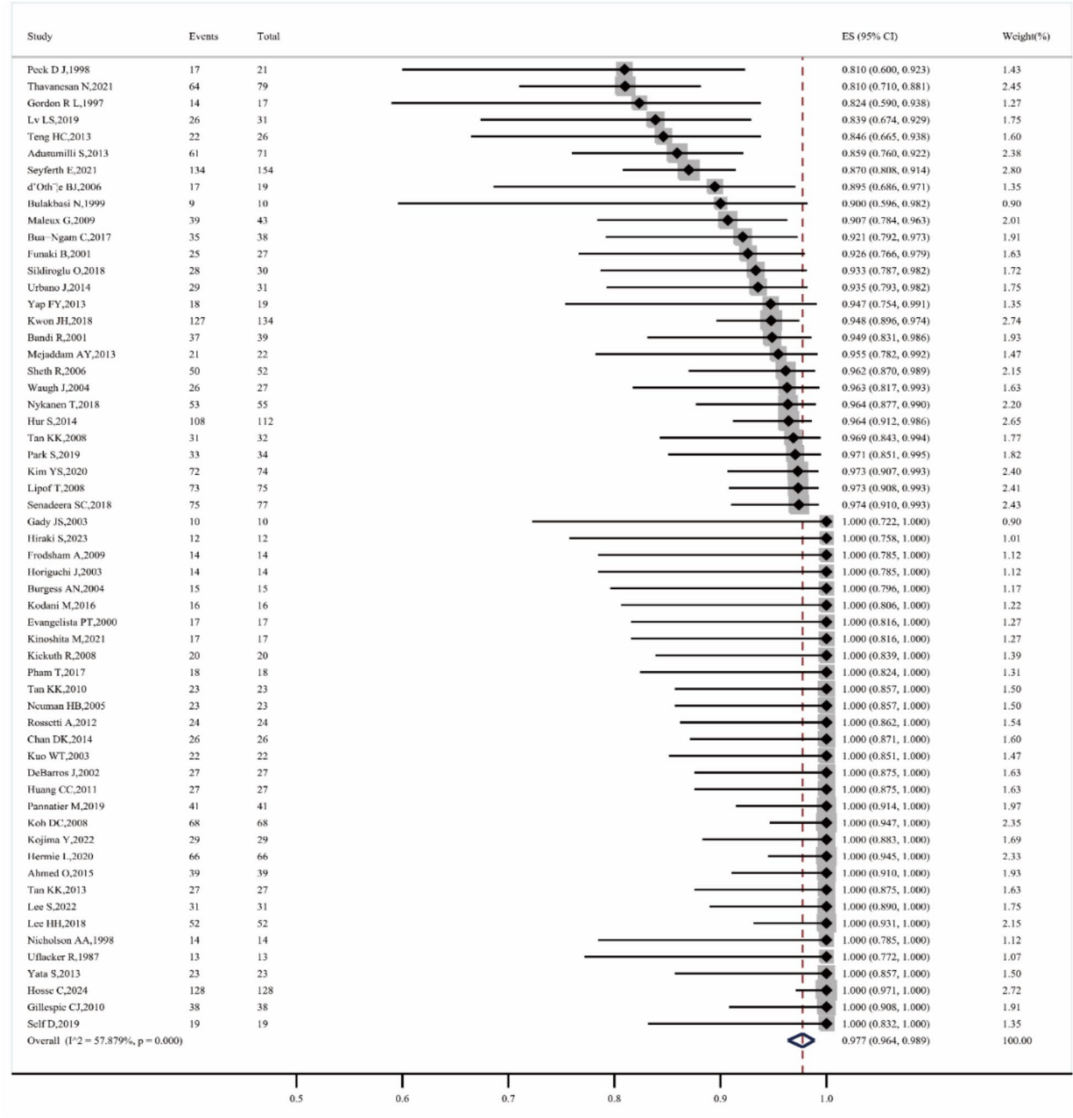
Forest plot of the technical success rate (I^2^ = 57.879%, p < 0.001)

**Fig. 3:**
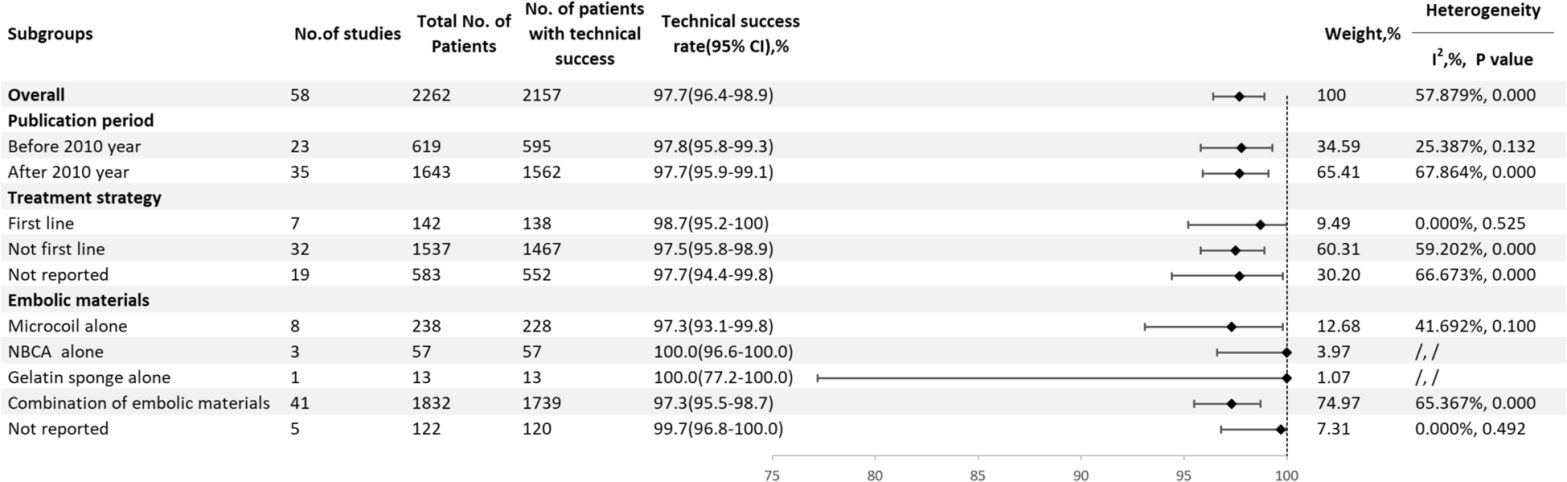
Result of subgroup meta–analyses for the technical success rate according to publication period, treatment strategy and embolic materials

A total of 58 studies reported the technical success rate [5, 9–14, 21–71], with an overall technical success rate of 97.7% (95% CI, 96.4–98.9, I^2^ = 57.879%, P < 0.001) (Fig. 2). Before 2010, the technical success rate was 97.8% (95% CI, 95.8–99.3, I^2^ = 25.387%, P = 0.132), while after 2010, it was 97.7% (95% CI 95.9–99.1, I^2^ = 67.864%, P < 0.001). The rate for first-line treatments was 98.7% (95% CI 95.2–100, I^2^ = 0.000%, P = 0.525), compared to 97.5% (95% CI 95.8–98.9, I^2^ = 59.202%,P < 0.001) for non–first–line treatments. When using microcoils alone, the technical success rate was 97.3% (95% CI 93.1–99.8, I^2^ = 41.692%, P = 0.100); for NBCA alone, it was 100.0% (95% CI 96.6–100.0); for Gelfoam alone, it was 100.0% (95% CI 77.2–100.0); and for mixed material embolization, it was 97.3% (95% CI 95.5–98.7, I^2^ = 65.367%, P < 0.001) (Fig. 3).

#### Clinical success rate

**Fig. 4:**
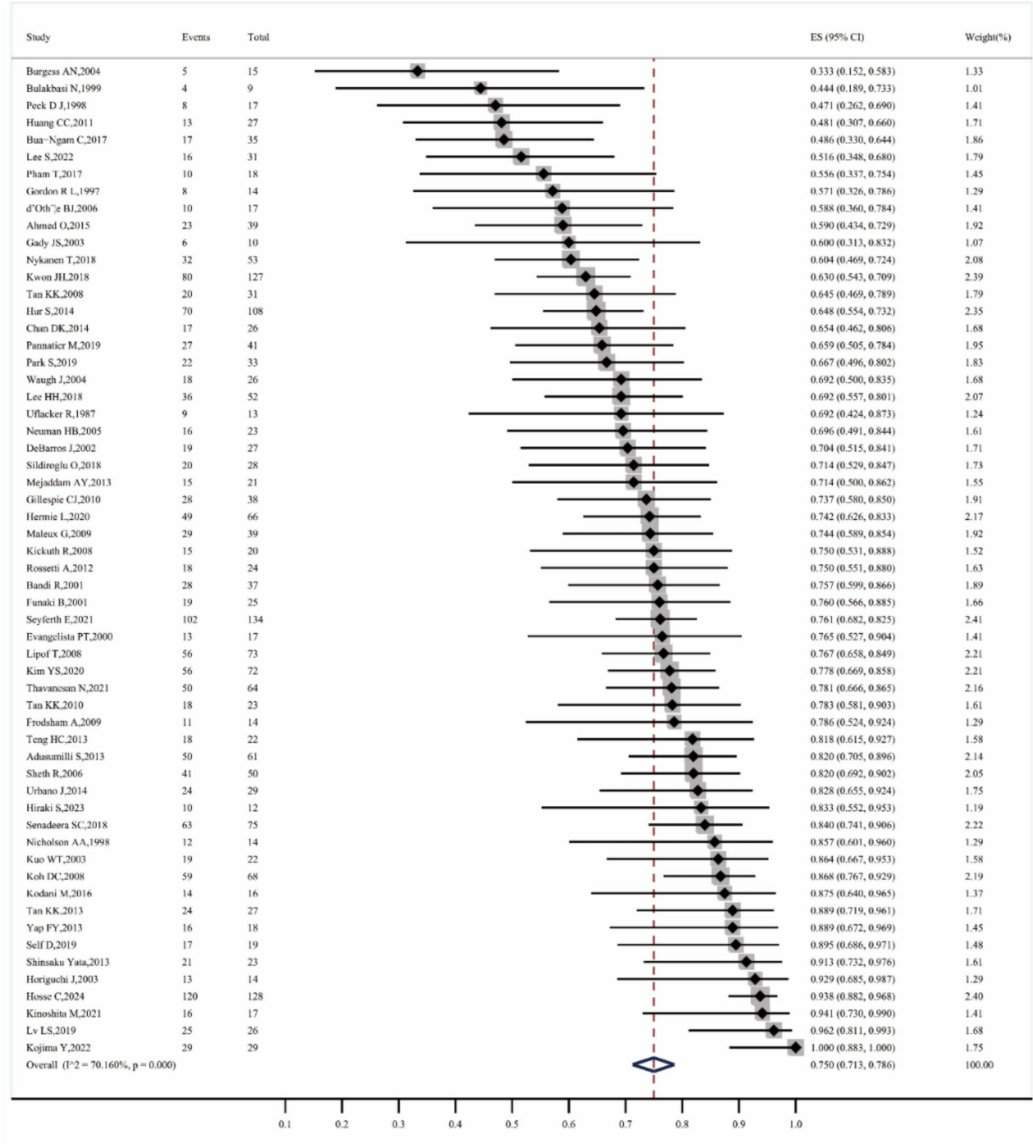
Forest plot of the clinical success rate (I^2^ = 70.160%, p < 0.001)

**Fig. 5:**
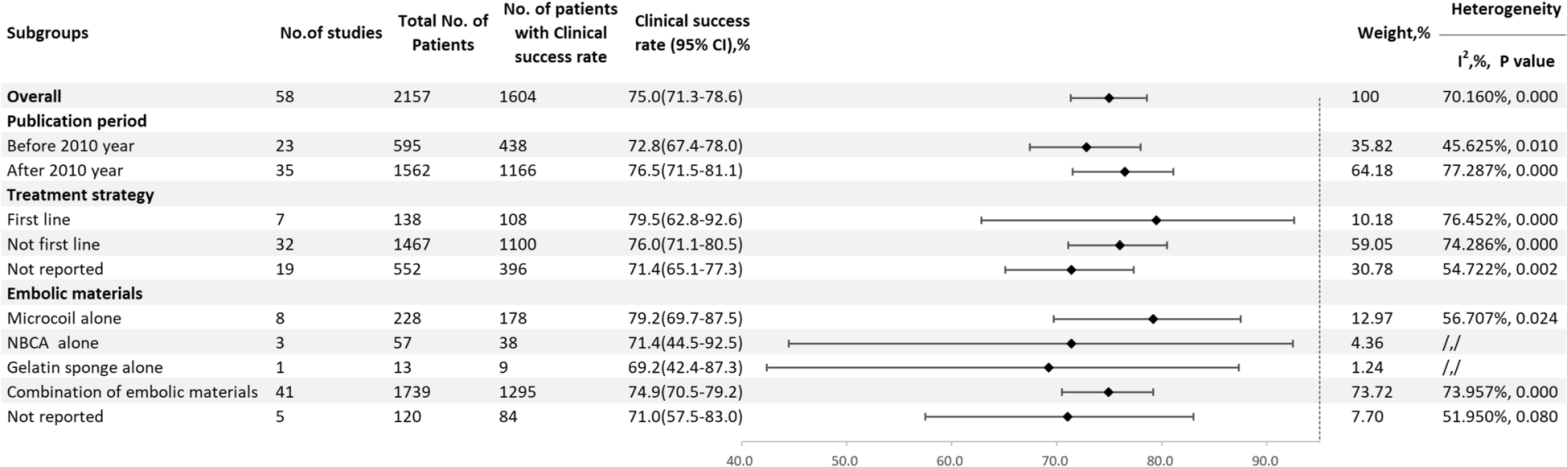
Result of subgroup meta–analyses for the clinical success rate according to publication period, treatment strategy and embolic materials

A total of 58 studies reported the clinical success rate [5, 9–14, 21–71], with an overall clinical success rate of 75.0% (95% CI 71.3–78.6, I^2^ = 70.160%, P < 0.001) (Fig. 4). Before 2010, the clinical success rate was 72.8% (95% CI 67.4–78.0, I^2^ = 45.625%, P = 0.010), while after 2010, it increased to 76.5% (95% CI 71.5–81.1, I^2^ = 77.287%, P < 0.001). For first–line treatments, the clinical success rate was 79.5% (95% CI 62.8–92.6, I^2^ = 76.452%, P < 0.001), compared to 76.0% (95% CI 71.1–80.5, I^2^ = 74.286%, P < 0.001) for non–first–line treatments. The clinical success rate for using microcoils alone was 79.2% (95% CI 69.7–87.5, I^2^ = 56.707%, P = 0.024), for NBCA alone it was 71.4% (95% CI 44.5–92.5), for Gelfoam alone it was 69.2% (95% CI 42.4–87.3), and for mixed material embolization, it was 74.9% (95% CI 70.5–79.2, I^2^ = 73.957%, P < 0.001) (Fig. 5).

#### Rebleeding rate

**Fig. 6:**
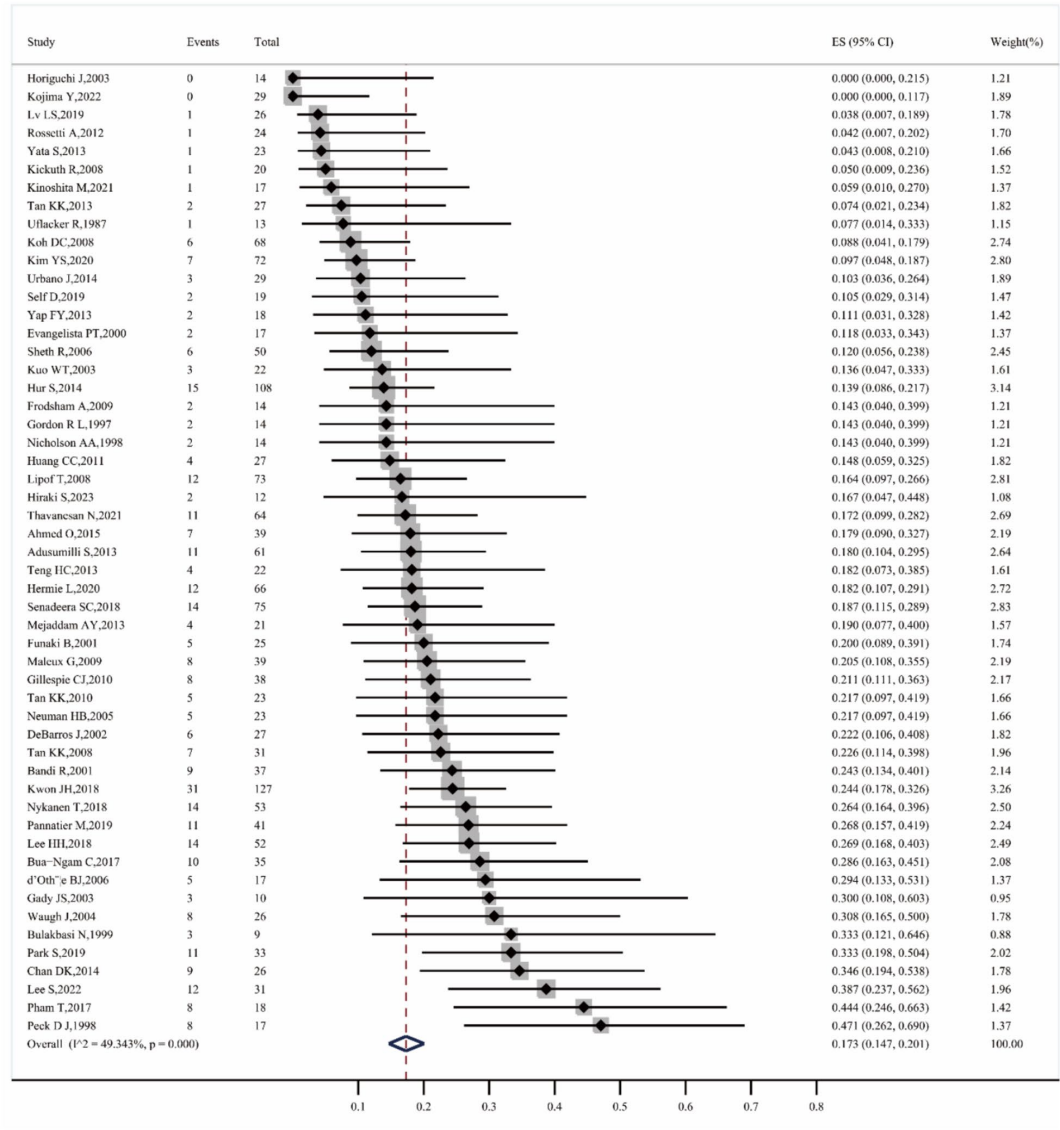
Forest plot of the re–bleeding rate (I^2^ = 49.343%, p < 0.001)

**Fig. 7:**
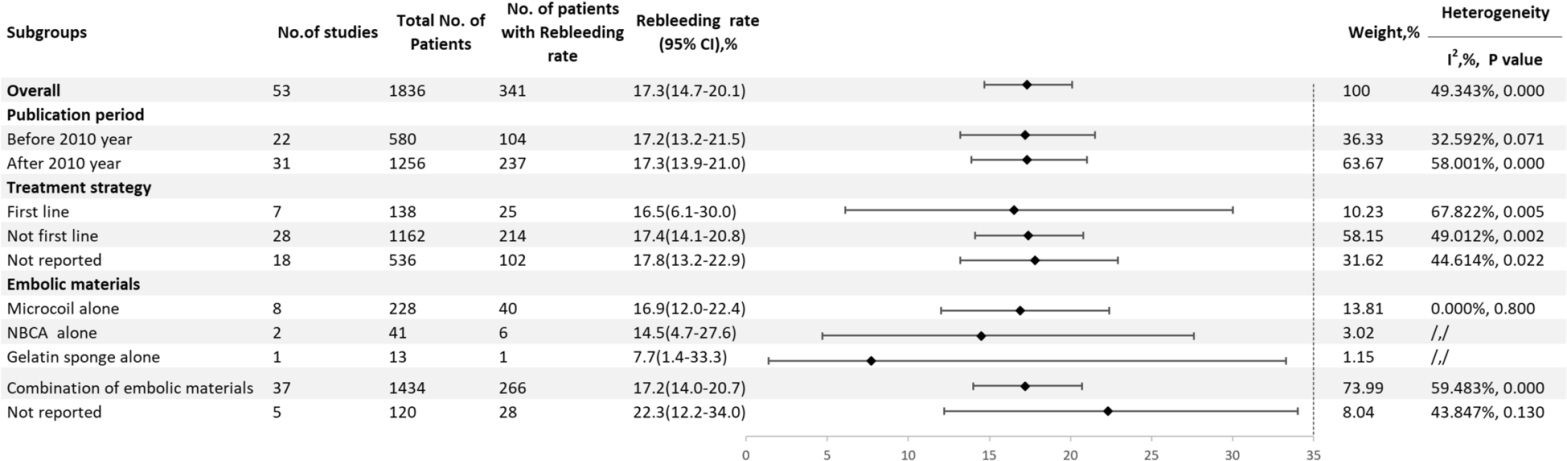
Result of subgroup meta–analyses for the re–bleeding rate according to publication period, treatment strategy and embolic materials

A total of 53 studies reported the rebleeding rate [5, 9–11, 13, 14, 21–25, 27–42, 45–52, 54–64, 66–71], with an overall rebleeding rate of 17.3% (95% CI 14.7–20.1, I^2^ = 49.343%, P < 0.001) (Fig. 6). The rebleeding rate before 2010 was 17.2% (95% CI 13.2–21.5, I^2^ = 32.592%, P = 0.071), while after 2010, it remained similar at 17.3% (95% CI 13.9–21.0, I^2^ = 58.001%, P < 0.001). For first–line treatments, the rebleeding rate was 16.5% (95% CI 6.1–30.0, I^2^ = 67.822%, P = 0.005), while for non–first–line treatments, it increased to 17.4% (95% CI 14.1–20.8, I^2^ = 49.012%, P = 0.002). The rebleeding rate for microcoils alone was 16.9% (95% CI 12.0–22.4, I^2^ = 0.000%, P = 0.800), for NBCA alone it was 14.5% (95% CI 4.7–27.6), for Gelfoam alone it was 7.7% (95% CI 1.4–33.3), and for mixed material embolization, it was 17.2% (95% CI 14.0–20.7, I^2^ = 59.483%, P < 0.001) (Fig. 7).

#### Secondary surgery rate

A total of 53 studies reported the secondary surgery rate [5, 9–11, 13, 14, 21–42, 44, 46–58, 60–67, 69–71], with an overall secondary surgery rate of 10.3% (95% CI 8.1–12.8, I^2^ = 55.707%, P < 0.001) (Supplementary Fig. 3). The secondary surgery rate before 2010 was 15.8% (95% CI 12.1–19.7, I^2^ = 24.001%, P = 0.151), while after 2010, it decreased to 7.7% (95% CI 5.4–10.3, I^2^ = 56.671%, P < 0.001). For first–line treatments, the secondary surgery rate was 5.5% (95% CI 1.2–11.8, I^2^ = 27.878%, P = 0.216), while for non–first–line treatments, the rate was higher at 10.9% (95% CI 8.5–13.5, I^2^ = 46.069%, P = 0.004). The secondary surgery rate for microcoils alone was 6.4% (95% CI 2.2–12.0, I^2^ = 40.109%, P = 0.111), for NBCA alone it was 4.1% (95% CI 0.0–17.0), for Gelfoam alone it was 38.5% (95% CI 17.7–64.5), and for mixed material embolization, the rate was 11.3% (95% CI 8.9–14.0, 52.011%, P < 0.001) (Supplementary Fig. 4).

#### Major complication

**Fig. 8:**
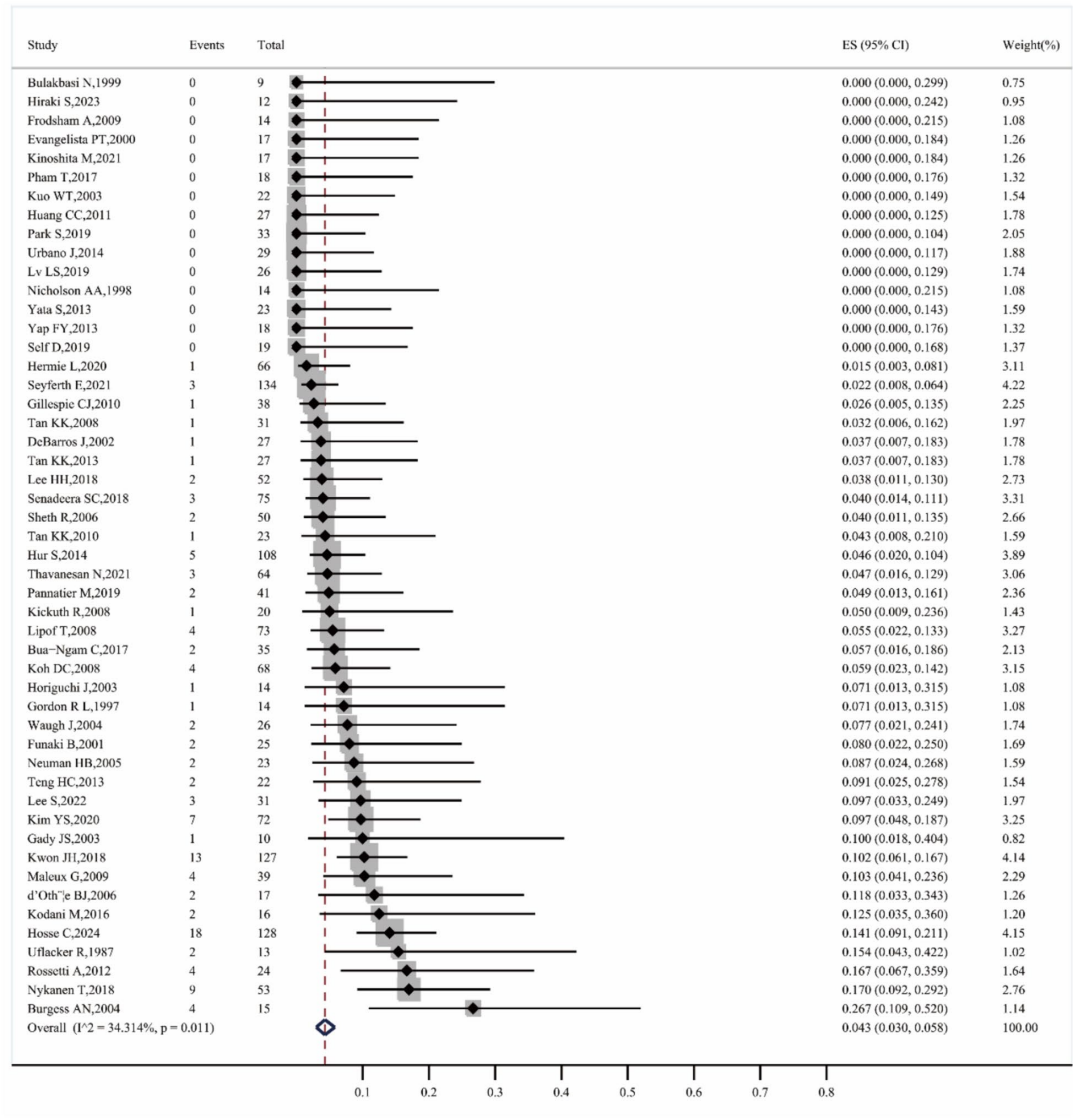
Forest plot of the major complications rate (I^2^ = 34.314%, p = 0.011)

**Fig. 9:**
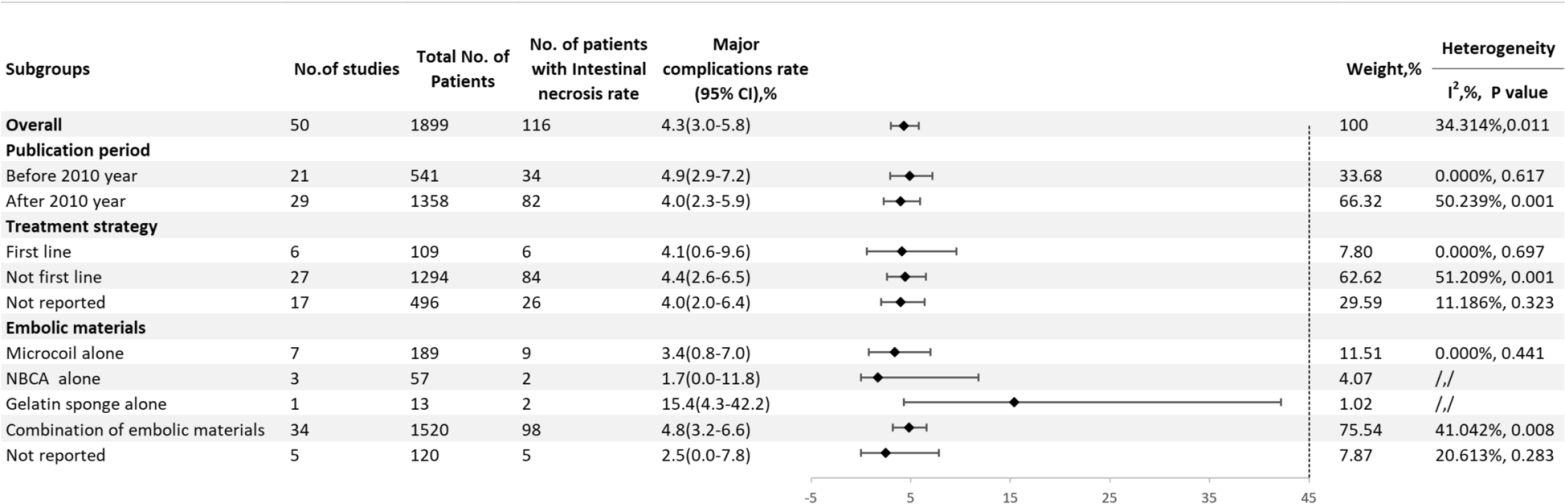
Result of subgroup meta–analyses for the major complications rate according to publication period, treatment strategy and embolic materials

A total of 50 studies reported the rate of major complications [5, 9–13, 21–35, 38–42, 44–46, 48, 49, 51–65, 67, 69–71], with an overall major complication rate of 4.3% (95% CI 3.0–5.8, I^2^ = 34.314%, P = 0.011) (Fig. 8). The major complication rate before 2010 was 4.9% (95% CI 2.9–7.2, I^2^ = 0.000%, P = 0.617), while after 2010, it decreased to 4.0% (95% CI 2.3–5.9, I^2^ = 50.239%, P = 0.001). For first–line treatments, the rate was 4.1% (95% CI 0.6–9.6, I^2^ = 0.000%, P = 0.697), while for non–first–line treatments, it was slightly higher at 4.4% (95% CI 2.6–6.5, I^2^ = 51.209%, P = 0.001). The rate for microcoils alone was 3.4% (95% CI 0.8–7.0, I^2^ = 0.000%, P = 0.441), for NBCA alone it was 1.7% (95% CI 0.0–11.8), for Gelfoam alone it was 15.4% (95% CI 4.3–42.2), and for mixed material embolization, the rate was 4.8% (95% CI 3.2–6.6, I^2^ = 41.042%, P = 0.008) (Fig. 9).

#### Mild ischemia rate

A total of 45 studies reported the incidence of mild ischemia [9–14, 21, 23–31, 33–36, 38–41, 43–48, 50, 51, 53–56, 58, 59, 62–64, 66, 67, 70, 71], with an overall rate of 4.7% (95% CI 2.9–6.7, I^2^ = 54.833%, P < 0.001) as shown in Supplementary Fig. 5. The rate of mild ischemia before 2010 was 7.4% (95% CI 3.9–11.6, I^2^ = 51.750%, P = 0.004), while it decreased to 3.3% (95% CI 1.7–5.4, I^2^ = 49.802%, P = 0.003) after 2010. In terms of treatment approaches, the incidence of mild ischemia for first–line treatment was 7.1% (95% CI 2.7–12.9, I^2^ = 5.021%, P = 0.389), whereas non–first–line treatments had a lower incidence of 4.3% (95% CI 2.4–6.7, I^2^ = 51.813%, P = 0.002). For specific embolization materials, the rates were as follows: microcoil alone 1.7% (95% CI 0.0–5.2, I^2^ = 28.519%, P = 0.201), NBCA alone 27.4% (95% CI 12.3–45.4), Gelfoam alone 0.0% (95% CI 0.0–22.8), and mixed embolization materials 5.1% (95% CI 3.1–7.4, I^2^ = 52.648%, P < 0.001), as illustrated in Supplementary Fig. 6.

#### Secondary complication

A total of 46 studies reported the incidence of secondary complications [9–14, 21, 23–31, 33–36, 38–41, 43–48, 50, 51, 53–56, 58–60, 62–64, 66, 67, 70, 71], with an overall incidence of 10.8% (95% CI 7.5–14.6, I^2^ = 76.844%, P < 0.001), as shown in Supplementary Fig. 7. The rate of secondary complications before 2010 was 17.9% (95% CI 10.8–26.3, I^2^ = 77.707%, P < 0.001), whereas it decreased to 7.2% (95% CI 4.2–10.8, I^2^ = 73.114%, P < 0.001) after 2010. Regarding treatment strategies, the incidence for first–line treatments was 17.2% (95% CI 5.7–32.3, I^2^ = 73.820%, P = 0.001), while non–first–line treatments had a lower incidence of 9.3% (95% CI 5.7–13.4, I^2^ = 73.990%, P < 0.001). When considering specific embolization materials, the rates of secondary complications were as follows: microcoil alone 3.2% (95% CI 0.3–7.9, I^2^ = 42.691%, P = 0.094), NBCA alone 27.4% (95% CI 12.3–45.4), Gelfoam alone 30.8% (95% CI 12.7–57.6), and mixed embolization materials 12.8% (95% CI 8.6–17.5, I^2^ = 79.124%, P < 0.001), as shown in Supplementary Fig. 8.

### Publication bias

Publication bias was assessed using funnel plots and Egger's linear regression test. Egger's test indicated no significant publication bias for the following outcomes: technical success rate (P = 0.098), clinical success rate (P = 0.348), rebleeding rate (P = 0.939), secondary operation rate (P = 0.233), major complications (P = 0.279), and mild ischemia rate (P = 0.723). In contrast, significant bias was detected for the rate of secondary complications (P = 0.039). The corresponding funnel plots are provided in Supplementary Figs. 9–15.

### Sensitivity analysis

To evaluate the robustness of our meta–analysis results and determine whether any individual study had an outsized influence on the overall findings, we performed a sensitivity analysis by sequentially excluding one study at a time. This procedure revealed only minor variations in the overall results, suggesting that our conclusions are both stable and reliable. The details of these analyses are presented in Supplementary Figs. 16–22.

## Discussion

A total of 58 studies on TAE for LGIB were included in this analysis, encompassing research findings on superselective embolization techniques from the 1980s onward. Studies with small sample sizes (< 10 cases) were excluded to enhance the breadth and robustness of the evidence. Outcome measures were uniformly defined to ensure the accuracy of the results. Meta–analysis of data from 2262 patients demonstrated that TAE achieved high clinical and technical success rates (75.0% and 97.7%, respectively) in the management of acute LGIB. The rebleeding rate was 17.3%, and major ischemia–related complications remained low at 4.3%. Rates of mild ischemia (4.7%) and other minor complications (10.8%) were also within an acceptable range.

Currently, research on TAE for LGIB consists predominantly of small–sample retrospective studies, with limited availability and quality of meta–analyses. Early meta–analytic findings reported an 85% success rate of embolization in diverticular bleeding, whereas for non–diverticular bleeding, the failure rate approached 45%  [72]. Two subsequent meta–analyses [73, 74] indicated that NBCA embolization achieved high technical success (97.8% and 98.8%) and clinical success rates (86.1% and 78.0%), accompanied by relatively low complication rates (major complications: 6.1% and 8.6%). The clinical success rate in the present study (75%) was somewhat lower than those reported in these earlier meta–analyses. This discrepancy may be attributed to our composite endpoint for clinical success, defined as immediate hemostasis without serious complications or mortality within 30 days, which likely resulted in a more conservative estimate of clinical success compared to prior studies [73, 74]. Nevertheless, this definition is considered to better capture the net clinical benefit of interventional therapy—beyond immediate technical success or short–term hemostasis. Even under this stringent criterion, TAE demonstrated a considerable pooled clinical success rate of 75.0%, underscoring its efficacy and safety as a key therapeutic option for LGIB.

In addition, the embolic materials used in these two studies were confined to NBCA and did not assess the impact of other embolic agents. In comparison to the aforementioned studies, the current analysis employed a wider array of embolic materials (e.g., microcoils, PVA particles, gelatin sponges), used singly or in combination, thereby facilitating a comprehensive evaluation of their safety and efficacy for acute LGIB. Moreover, the three earlier meta–analyses incorporated only 5–6 LGIB articles each, with limited sample sizes and considerable heterogeneity in study quality, thereby constraining the robustness and generalizability of their conclusions.

The analysis revealed substantial heterogeneity in the pooled rates of technical success (I^2^ = 57.879%), clinical success (I^2^ = 70.160%), secondary surgery (I^2^ = 55.707%), mild ischemia (I^2^ = 54.833%), and secondary complications (I^2^ = 76.844%) following TAE for LGIB, indicating considerable variation across studies. To investigate potential sources of this heterogeneity, subgroup analyses were performed. These analyses demonstrated an overall high technical success rate (97.7%) with minimal variation among subgroups. Higher clinical success rates were observed in subgroups from after 2010 (76.5%), those receiving TAE as first–line treatment (79.5%), and those embolized with microcoils (79.2%). Notably, heterogeneity in technical and clinical success rates was most pronounced in the post–2010 subgroup (I^2^ = 67.864%; I^2^ = 77.287%) and the mixed embolization materials subgroup (I^2^ = 65.367%; I^2^ = 73.957%).

Furthermore, the incidence of secondary surgery, mild ischemia, and secondary complications showed varying degrees of reduction after 2010. Specifically, the secondary surgery rate decreased from 15.8% (95% CI 12.1–19.7%) to 7.7% (95% CI 5.4–10.3%), the mild ischemia rate declined from 7.4% (95% CI 3.9–11.6%) to 3.3% (95% CI 1.7–5.4%), and the secondary complication rate fell from 17.9% (95% CI 10.8–26.3%) to 7.2% (95% CI 4.2–10.8%). However, substantial heterogeneity persisted for these three outcomes in the mixed embolic materials subgroup (I^2^ = 52.011%, 52.648%, and 79.124%, respectively). High heterogeneity was also observed for the secondary surgery rate in the post–2010 subgroup (I^2^ = 56.671%) and for secondary complications in subgroups around 2010 (I^2^ = 77.707%; I^2^ = 73.114%).

Based on these results, the authors speculate that the heterogeneity may be attributed to variations among medical centers after 2010 in the selection of embolic materials, the application of DSA navigation systems, and implementation of novel techniques. In earlier periods, the selection of embolic agents was relatively limited, whereas modern embolic materials have been developed to enable more precise, controllable, and safe vascular occlusion. Nevertheless, the high heterogeneity observed with the combined use of embolic materials may reflect the flexible and individualized application of various agents in clinical practice, leading to considerable variation across outcome measures. Additionally, the stringent criteria adopted for defining clinical success—incorporating both immediate hemostasis and the absence of serious complications or mortality within 30 days—may introduce greater clinical variability into the assessment.

Since 2010, key outcome measures—including rates of secondary surgery, mild ischemia, and secondary complications—have shown consistent improvement. These findings reflect substantial advances in embolic materials and interventional imaging technology over the past decade. The ongoing development of diverse microcoils—such as bare metal, fibered, protein–coated, polymer–coated, and shape–memory polymer coils—along with improved controlled–release designs, allows operators to confirm optimal positioning before deployment, enhancing procedural accuracy and safety while minimizing risks of non–target embolization and bowel ischemia. In recent years, standardized gelatin sponges with uniform particle sizes have provided more predictable embolization levels, reducing the likelihood of capillary bed occlusion and subsequent intestinal infarction. Furthermore, the radiopacity and polymerization rate of NBCA can be modulated by adjusting its mixture with lipiodol or tantalum powder, optimizing its use in actively bleeding vessels for rapid hemostasis [75].

At the same time, advancements in image–guided technology form the cornerstone of precision embolization, as exemplified by cone–beam CT (CBCT). This technology elevates interventional navigation by enabling the DSA system to rotate around the patient during the procedure, acquiring CT–like three–dimensional volumetric data. In the context of LGIB embolization, CBCT facilitates precise anatomical localization and clearly delineates the spatial relationships between the intestinal tract and its vasculature. This capability aids in identifying the culprit bleeding vessel, detecting occult hemorrhage, and allows for fusion with DSA images to provide real–time guidance—thereby enhancing procedural success and reducing operation time [48, 76].

In this analysis, both the rebleeding rate and major complication rate (intestinal infarction) exhibited low heterogeneity. Nevertheless, the prespecified subgroup analyses yielded valuable insights. Estimates for these outcomes demonstrated high consistency across all subgroups, reflecting stable treatment effects under diverse clinical conditions and reinforcing the robustness of the primary findings.

In addition, publication bias was assessed using funnel plots and Egger’s test. The results indicated the presence of publication bias for the incidence of secondary complications (Egger’s test, p = 0.039; see Supplementary Fig. 15 for funnel plots). Asymmetry in the funnel plot suggests that "negative" studies reporting higher complication rates may have remained unpublished. This bias likely arises from the "positive outcome bias" prevalent in academic publishing, wherein studies demonstrating the safety and efficacy of a technique are more likely to be published than those reporting complications. Moreover, a tendency within interventional radiology to highlight technical successes may further exacerbate this issue. These factors may have led to an underestimation of the true incidence of secondary complications in the present analysis.

The 2021 Imaging Guidelines [7] recommend an individualized approach to managing recurrent LGIB, particularly in hemodynamically unstable patients, suggesting that the choice between repeat TAE, emergency endoscopy, or surgery be guided by bleeding location and etiology. In clinical practice, nutritional status serves as a key factor when formulating personalized treatment strategies for LGIB patients. Evidence indicates that malnutrition contributes to impaired multi–organ function, manifesting systemically as reduced muscle strength and diminished cardiorespiratory reserve. Chronic nutritional deficits may further compromise intestinal perfusion, mucosal integrity, coagulation mechanisms, and immune function, thereby significantly elevating the risk of adverse clinical outcomes. Compared to well–nourished individuals, malnourished patients exhibit markedly higher rates of postoperative complications—such as pneumonia, surgical site infection, and anastomotic leakage—as well as increased mortality. Additionally, nutrition–related coagulation dysfunction may raise the risk of rebleeding or puncture site hematoma following interventional procedures. Therefore, early nutritional risk assessment and proactive, standardized nutritional support throughout the management of acute LGIB are clinically essential to mitigate serious complications and reduce hospital stay, particularly in high–risk and frail populations [77, 78].

This study’s major advantage lies in its comprehensive review of the existing literature on TAE for LGIB, combined with the harmonization and standardization of key outcome measures, notably through precise definitions of technical and clinical success. The exclusion of data pertaining to iatrogenic bleeding embolization helped minimize heterogeneity, thus enhancing the precision and comparability of the results. However, several limitations should be noted. First, the study protocol was not prospectively registered on international platforms such as PROSPERO, and all included studies were retrospective, potentially introducing selection bias. The exclusion of gray literature may also have contributed to publication bias, which was observed for secondary complication outcomes. Second, considerable heterogeneity was present across studies. Third, the incidence of ischemic complications may be underestimated, as some studies did not perform routine early endoscopic evaluation after TAE. Fourth, although embolic material selection critically influences outcomes, most studies employed mixed agents, preventing independent analysis of specific materials and limiting generalizability. Finally, the predominance of uncontrolled retrospective case series resulted in evidence of relatively low quality. Prospective randomized controlled trials remain necessary to validate these findings.

## Conclusion

This study provides a comprehensive meta–analysis of the existing literature on TAE for the treatment of LGIB. It confirms that TAE is an effective treatment modality for LGIB, demonstrating not only high technical and clinical success rates, but also an acceptable incidence of re–bleeding and ischemic complications. Despite some heterogeneity and limitations in the current evidence, TAE’s potential in managing LGIB is widely acknowledged. Future high–quality prospective studies and multi–center randomized controlled trials are essential to further validate the efficacy, safety, and applicability of TAE across diverse patient populations.

## Supplementary Information

Supplementary Material 1.

Supplementary Material 2.

## Data Availability

No datasets were generated or analysed during the current study.
