# Supplementary material for: Efficacy and safety of transcatheter arterial embolization for lower gastrointestinal bleeding: a systematic review and meta-analysis of 58 clinical trials
Source: Eur J Med Res. 2025 Dec 2;31:33. doi: 10.1186/s40001-025-03605-0 (PMC12776983; doi:10.1186/s40001-025-03605-0)
Supplement: Supplementary file 1 — Supplementary Material 1. [file 40001_2025_3605_MOESM1_ESM.docx]

**Search strategy**

**1 Detailed Search Strategy for PubMed**

("Lower Gastrointestinal tract "[Mesh] OR (lower gastrointestin*[Title/Abstract] OR lower GI[Title/Abstract] OR colonic [Title/Abstract] OR colorectal [Title/Abstract] OR intestin*[Title/Abstract])) AND

(("Hemorrhage"[Mesh] OR "Gastrointestinal Hemorrhage"[Mesh]) OR (bleed*[Title/Abstract] OR hemorrhag*[Title/Abstract] OR haemorrhag*[Title/Abstract])) AND

(("Angiography"[Mesh] OR "Radiography, Interventional"[Mesh]) OR (catheter angiograph*[Title/Abstract] OR transarterial [Title/Abstract] OR trans-arterial [Title/Abstract]))

**2 Detailed Search Strategy for Embase**

#1 'lower gastrointestinal tract' OR 'intestine disease' OR 'gastrointestinal hemorrhage' OR 'lower gastrointestin' OR 'lower gi' OR 'colonic' OR 'colorectal' OR 'intestin' OR 'rect' OR 'sigmoid':ti,ab,kw

#2 'hemorrhage' OR 'gastrointestinal hemorrhage' OR 'bleed' OR 'hemorrhag' OR 'haemorrhag' OR 'blood loss':ti,ab,kw

#3'angiography' OR 'interventional radiology' OR 'arterial embolization' OR 'catheter angiograph' OR 'transarterial' OR 'trans-arterial' OR 'arterial embol' OR 'embolotherap':ti,ab,kw

#1 AND #2 AND #3

**3 Detailed Search Strategy for Web of science**

(("lower gastrointestin*" OR "lower gi" OR colonic OR colorectal OR intestin* OR rect* OR sigmoid) AND

(bleed* OR hemorrhag* OR haemorrhag* OR "blood loss")) AND

("catheter angiograph*" OR transarterial OR "trans-arterial" OR "arterial embol*" OR embolotherap*)

**4 Detailed Search Strategy for Cochrane**

"Lower gastrointestinal tract" OR "intestine disease" OR "gastrointestinal hemorrhage" OR "lower gastrointestin" OR "lower gi" OR "colonic" OR "colorectal" OR "intestin" OR "rect" OR "sigmoid"

AND "hemorrhage" OR "gastrointestinal hemorrhage" OR "bleed" OR "hemorrhag" OR "haemorrhag" OR "blood loss"

AND "angiography" OR "interventional radiology" OR "arterial embolization" OR "catheter angiograph" OR "transarterial" OR "trans-arterial" OR "arterial embol" OR "embolotherap"
