# Supplementary material for: Efficacy and safety of transcatheter arterial embolization for lower gastrointestinal bleeding: a systematic review and meta-analysis of 58 clinical trials
Source: Eur J Med Res. 2025 Dec 2;31:33. doi: 10.1186/s40001-025-03605-0 (PMC12776983; doi:10.1186/s40001-025-03605-0)
Supplement: Supplementary file 2 — Supplementary Material 2. [file 40001_2025_3605_MOESM2_ESM.docx]

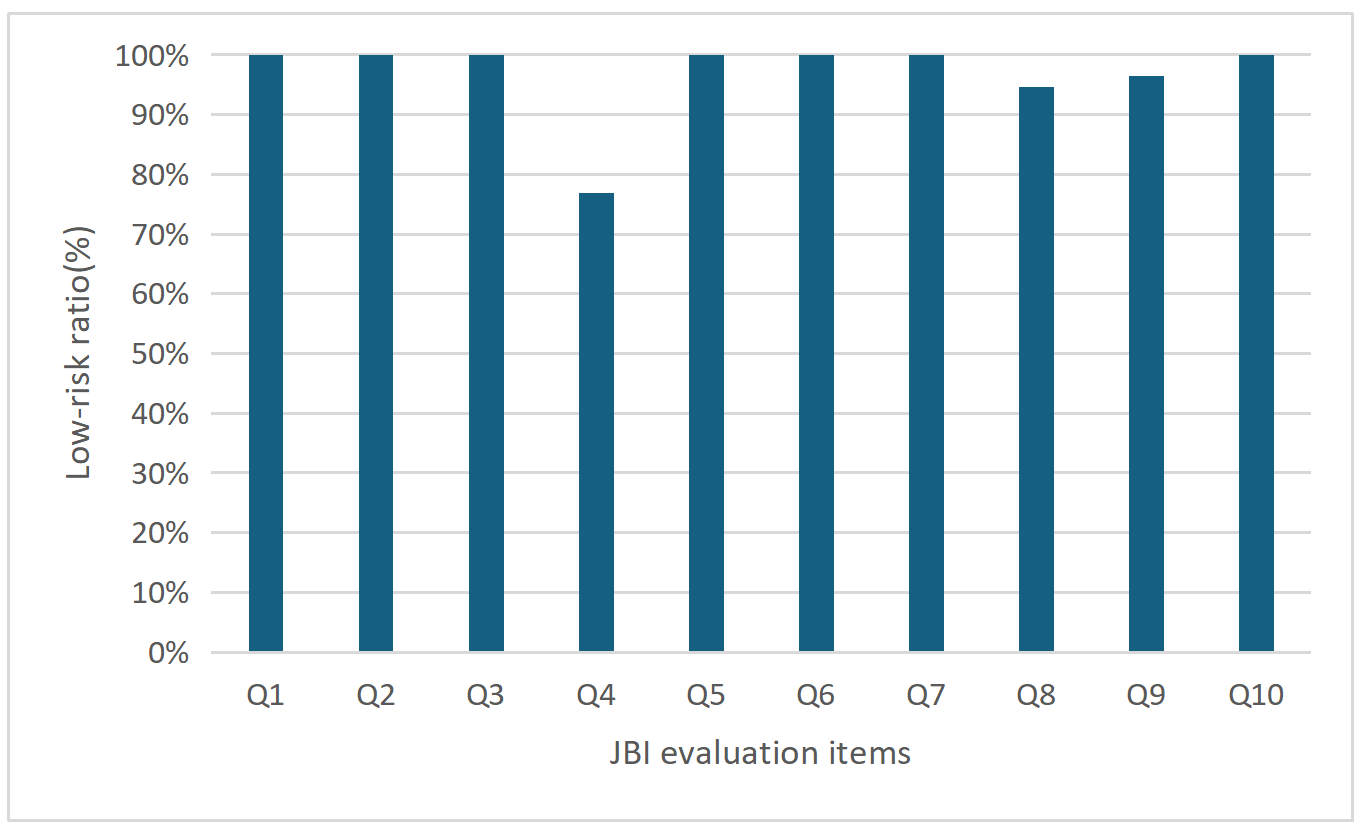


Supplementary Fig 1. Proportion of studies rated as ‘low risk of bias’ for each item of the JBI critical appraisal checklist.


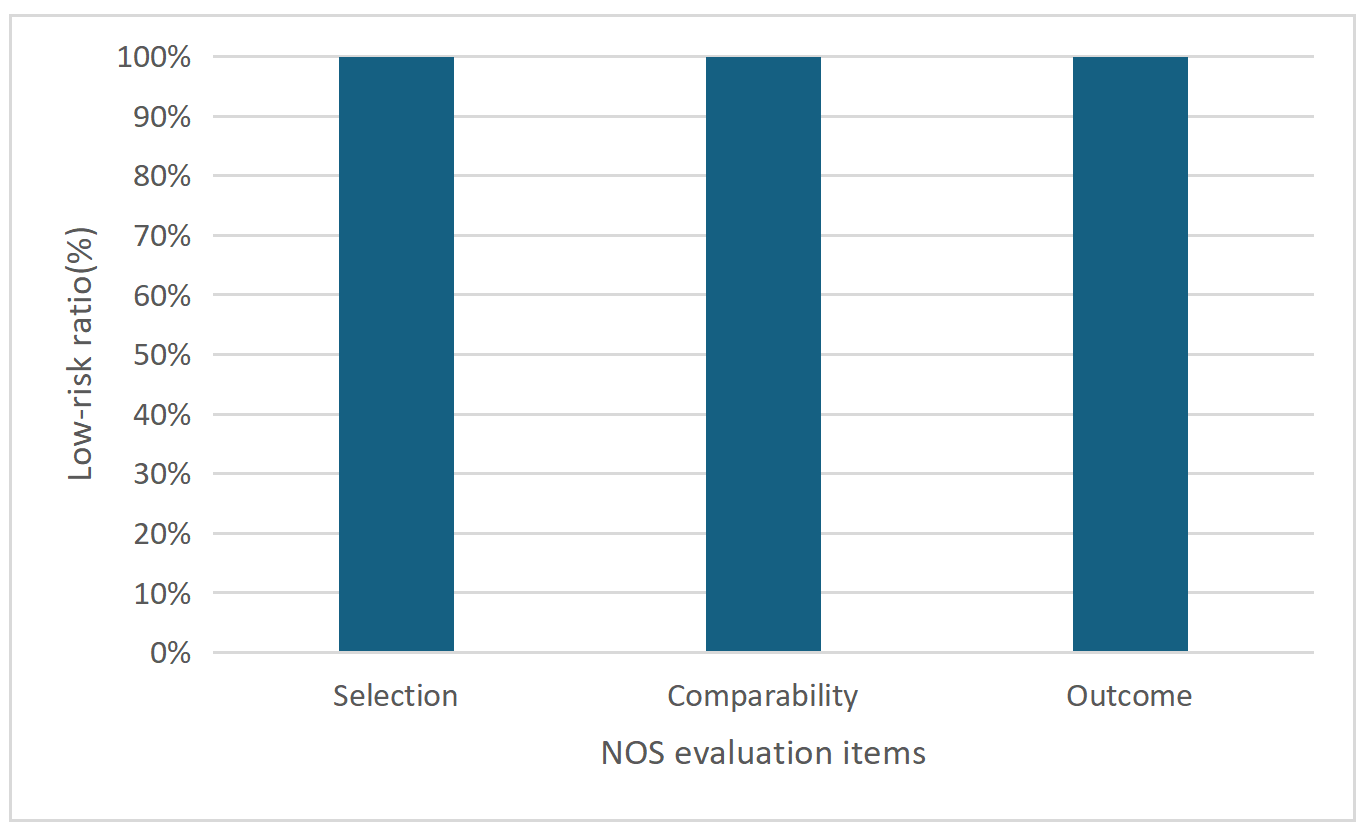


Supplementary Fig 2. Proportion of studies rated as ‘low risk of bias’ for each domain of the Newcastle-Ottawa Scale.


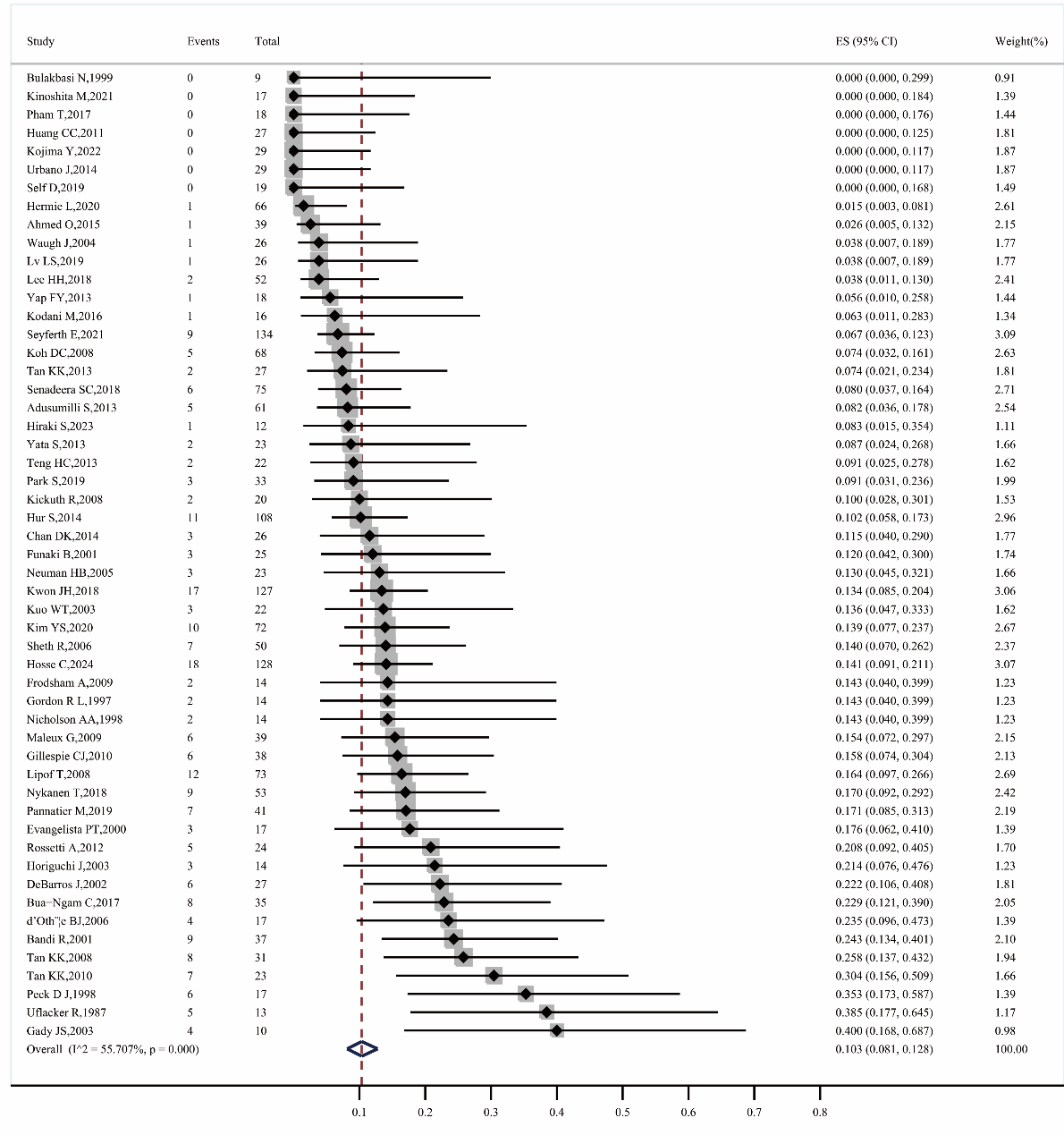


Supplementary Fig 3. Forest plot of the secondary surgery rate (I² = 55.707%, p < 0.001).


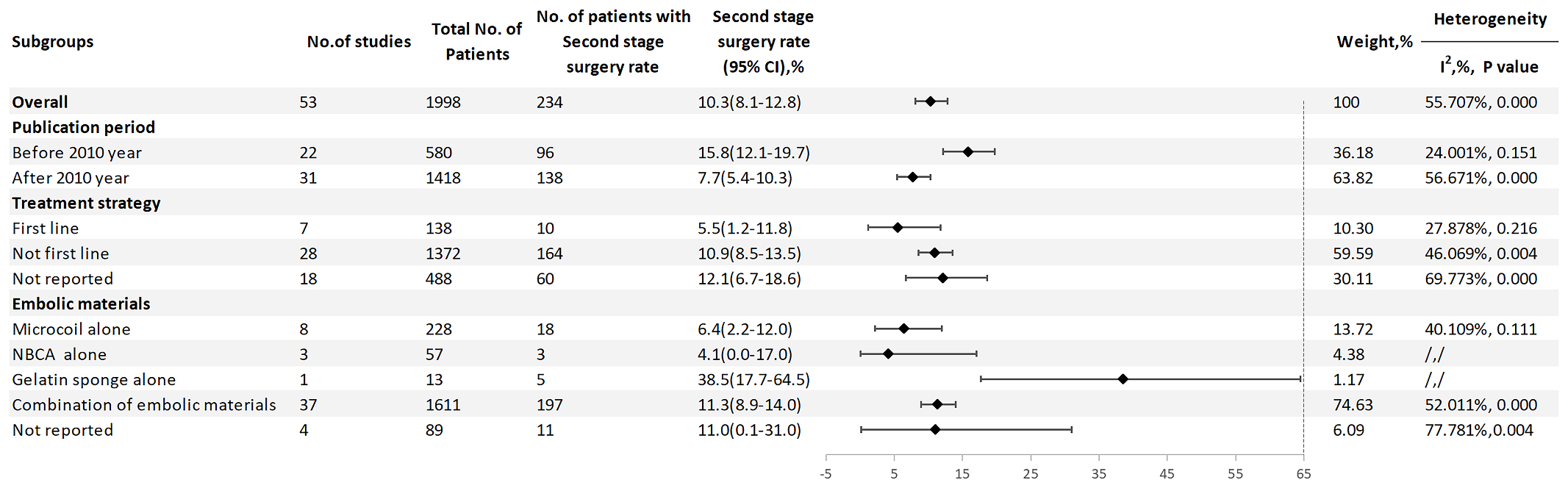


Supplementary Fig 4. Result of subgroup meta-analyses for secondary surgery rate according to publication period, treatment strategy and embolic materials.


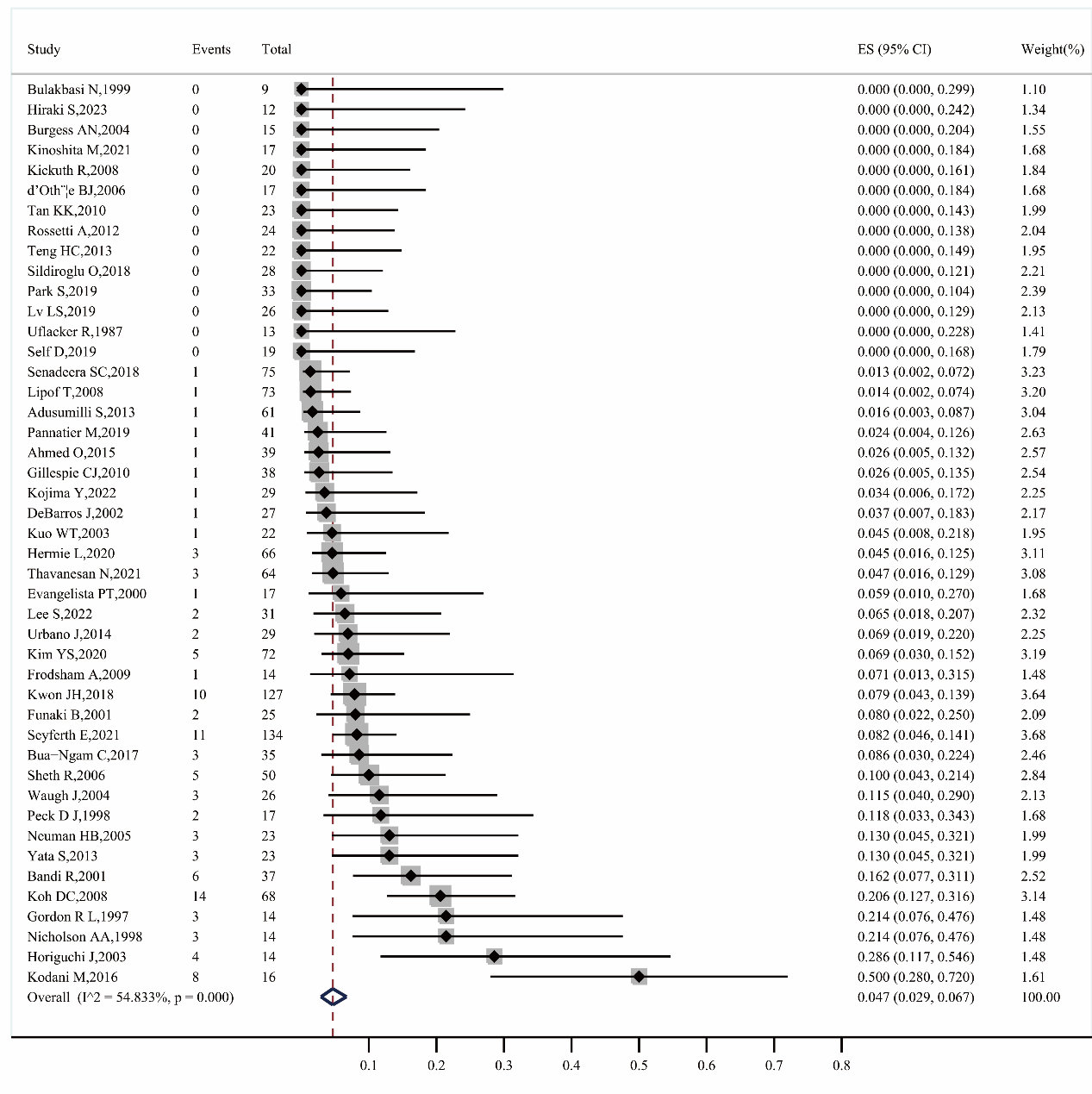


Supplementary Fig 5. Forest plot of the mild ischemia rate (I² = 54.833%, p < 0.001).


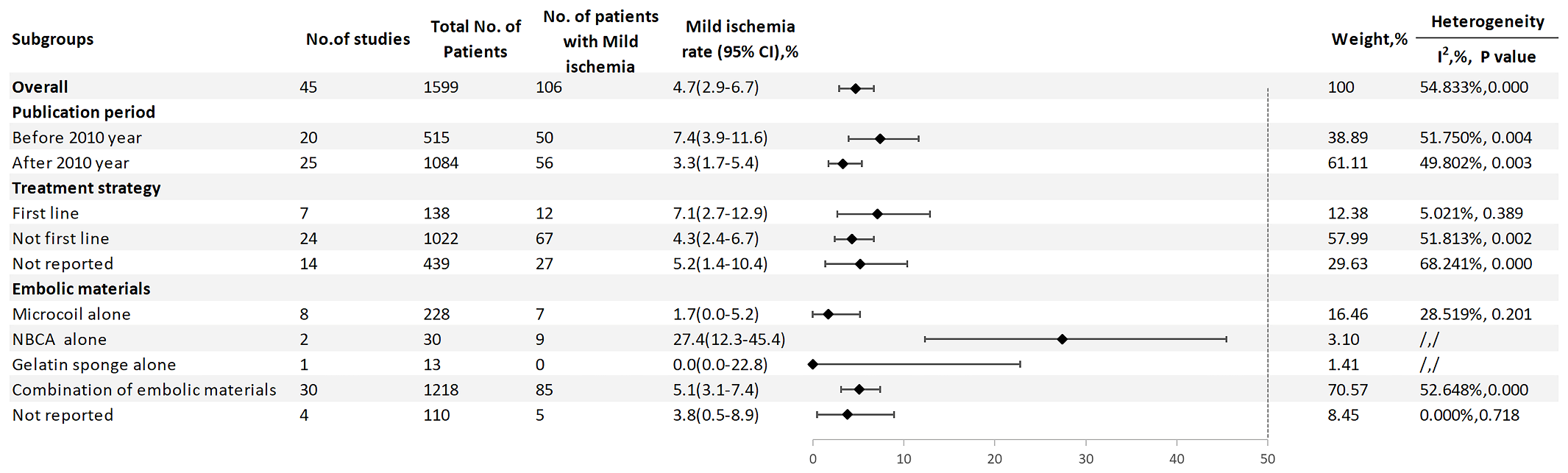


Supplementary Fig 6. Result of subgroup meta-analyses for mild ischemia rate according to publication period, treatment strategy and embolic materials.
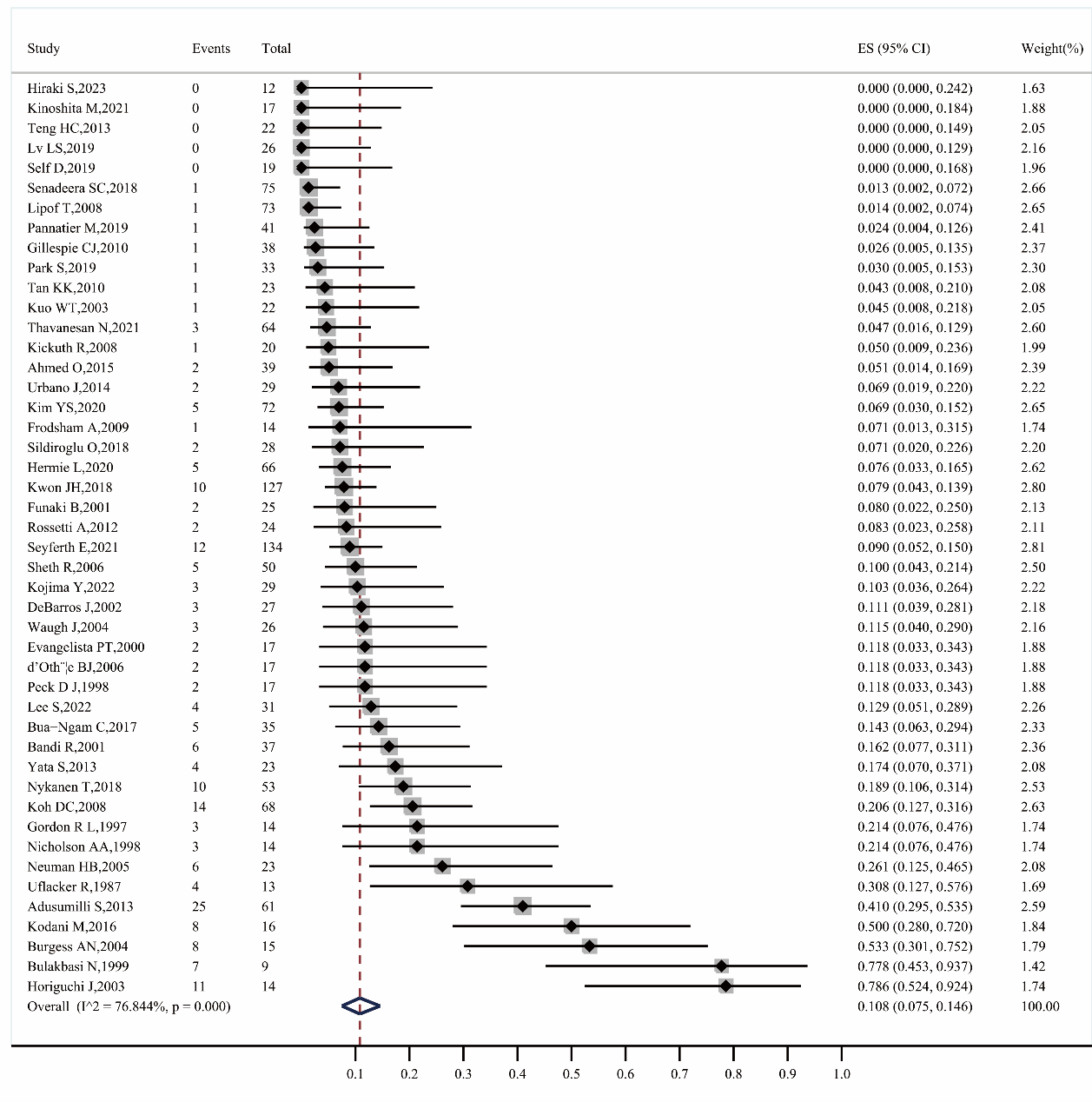


Supplementary Fig 7. Forest plot of the secondary complications rate (I² = 76.844%, p < 0.001).


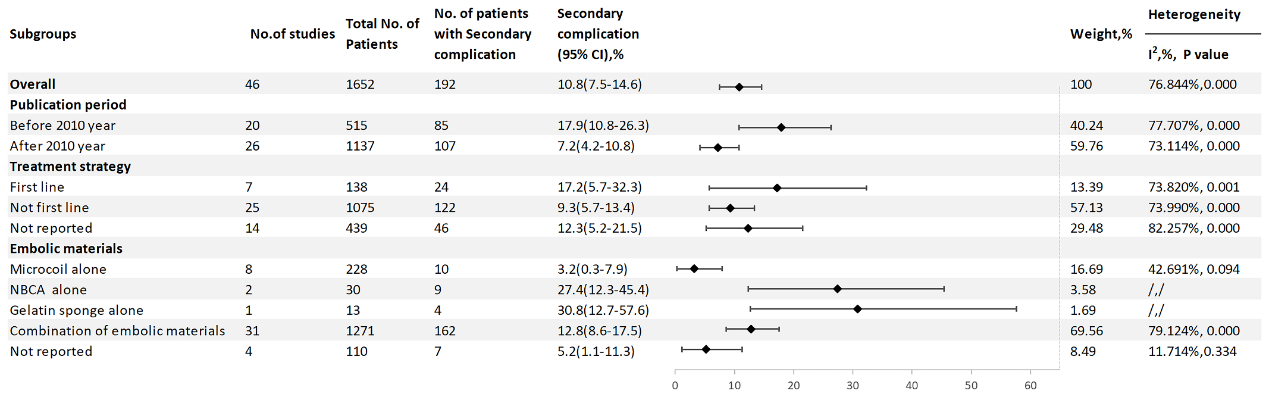


Supplementary Fig 8. Result of subgroup meta-analyses for secondary complications rate according to publication period, treatment strategy and embolic materials.


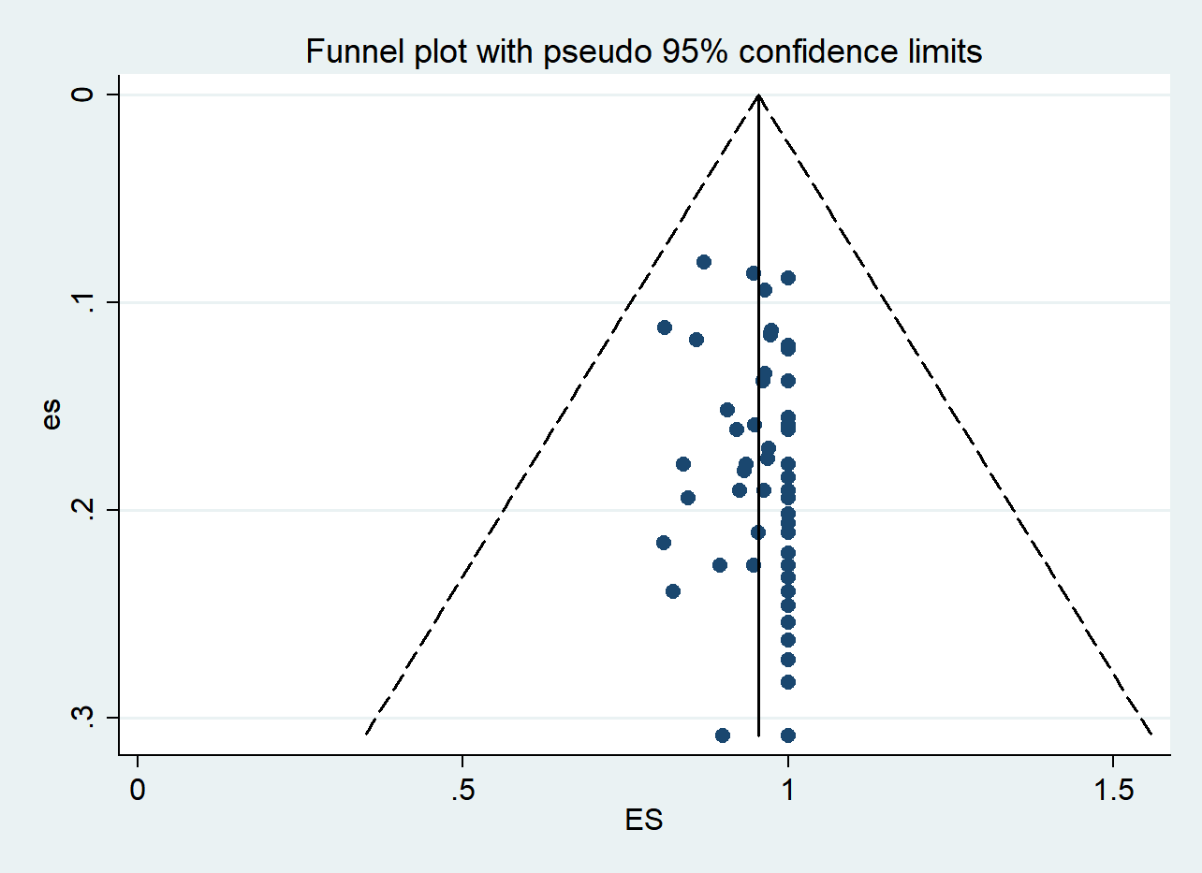


Supplementary Fig 9. Funnel plot of publication bias for technical success.


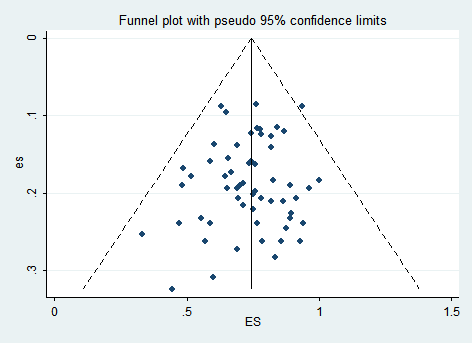


Supplementary Fig 10. Funnel plot of publication bias for clinical success.


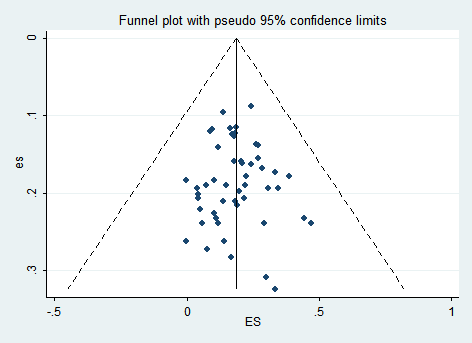


Supplementary Fig 11. Funnel plot of publication bias for re-bleeding.


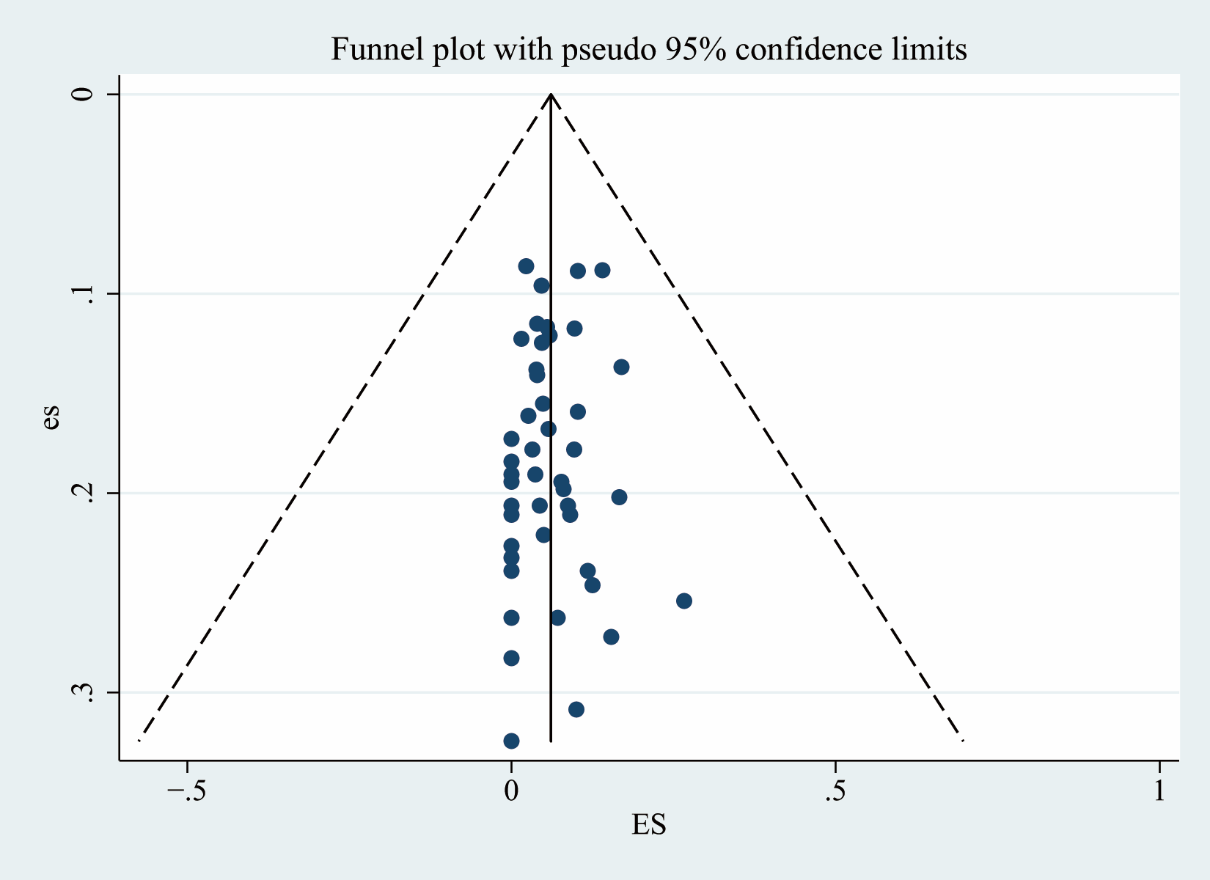


Supplementary Fig 12. Funnel plot of publication bias for major complications.


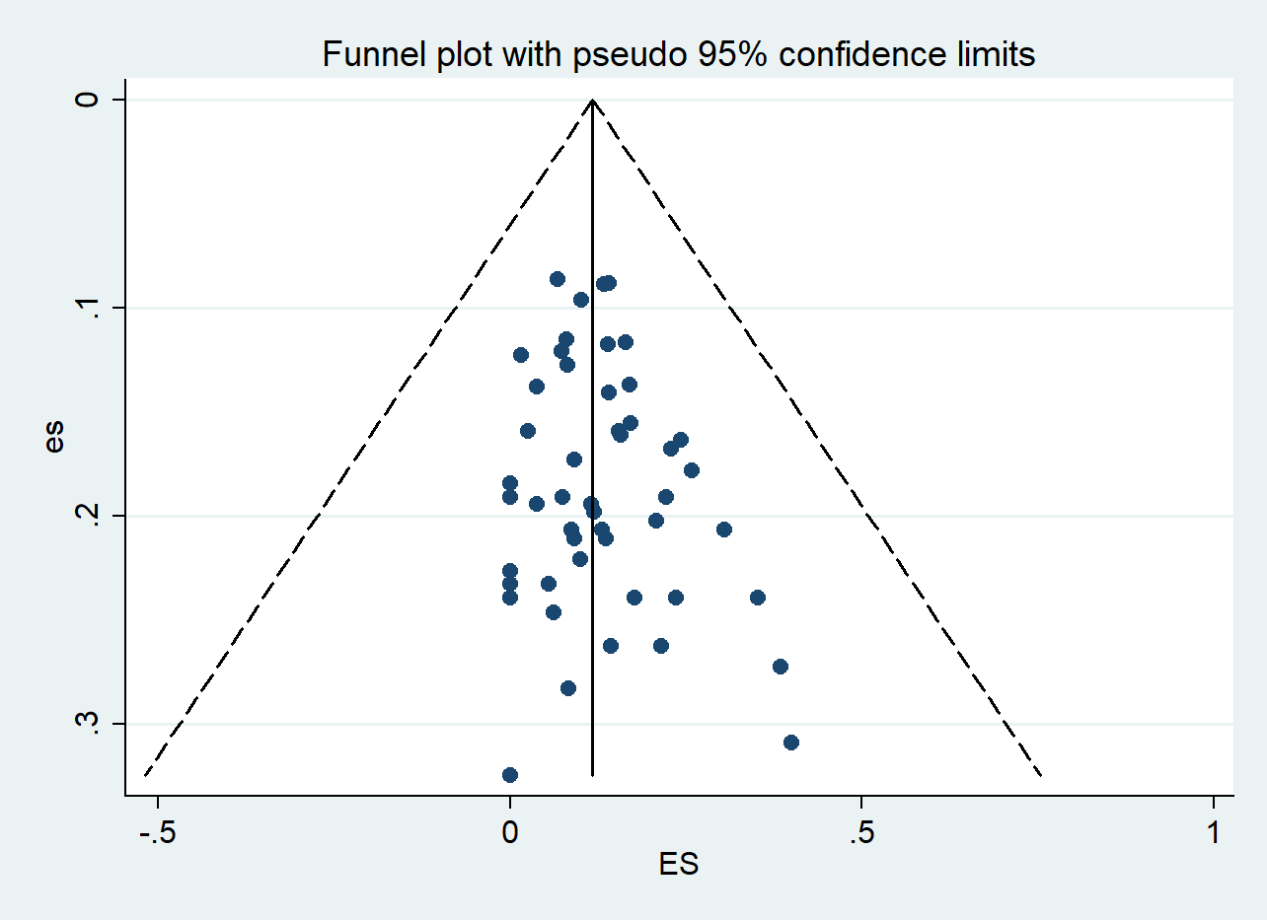


Supplementary Fig 13. Funnel plot of publication bias for secondary surgery.


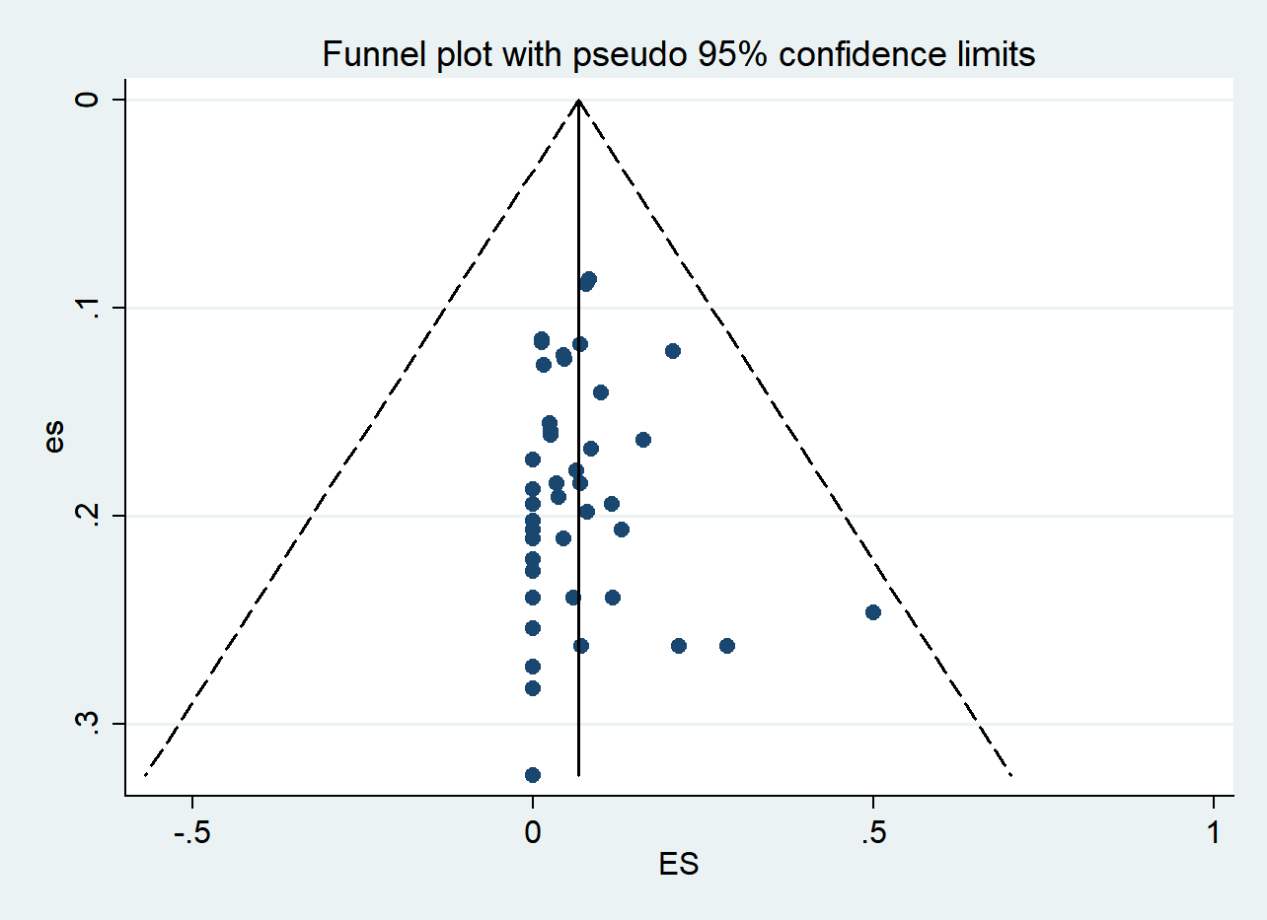


Supplementary Fig 14. Funnel plot of publication bias for mild ischemia.


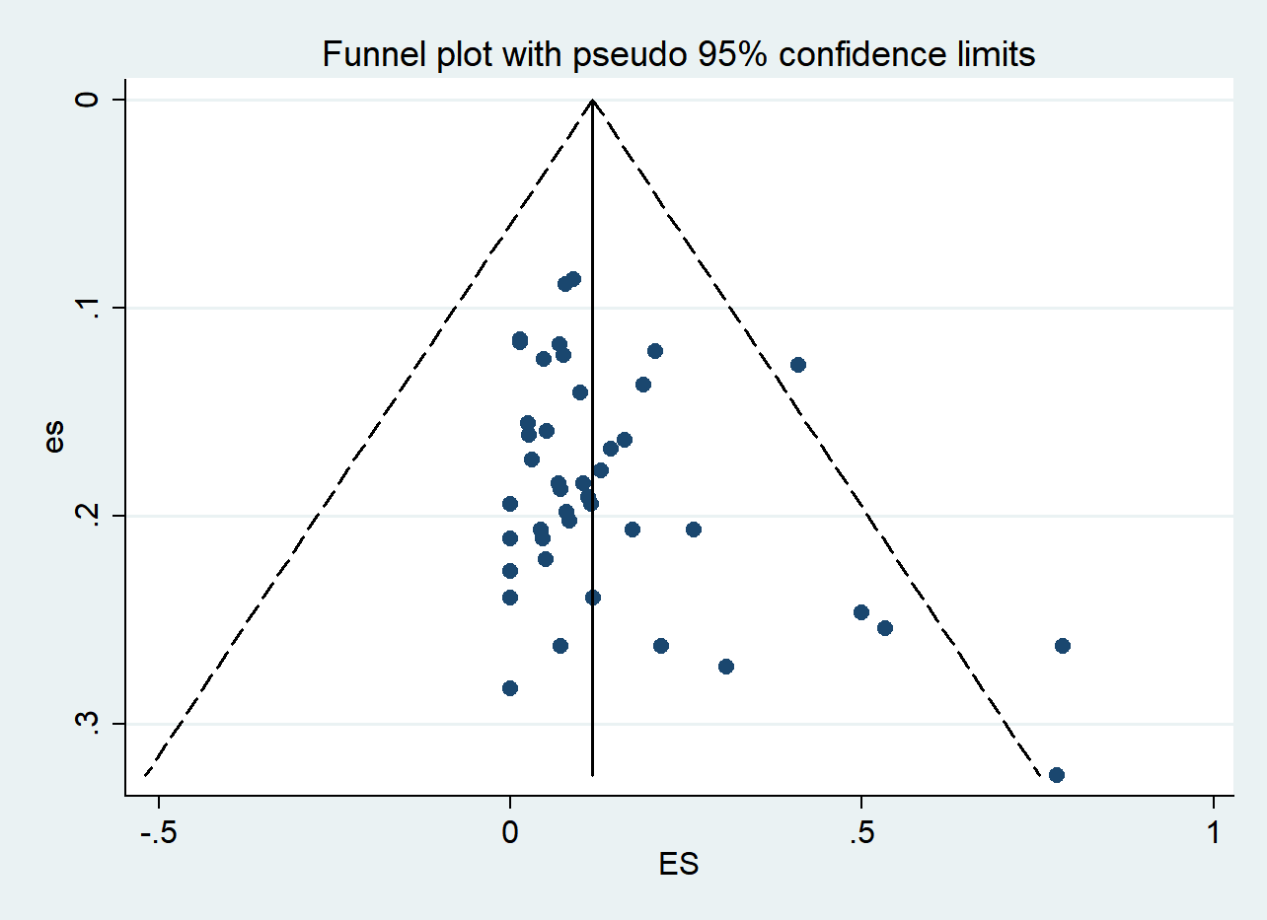


Supplementary Fig 15. Funnel plot of publication bias for secondary complications.


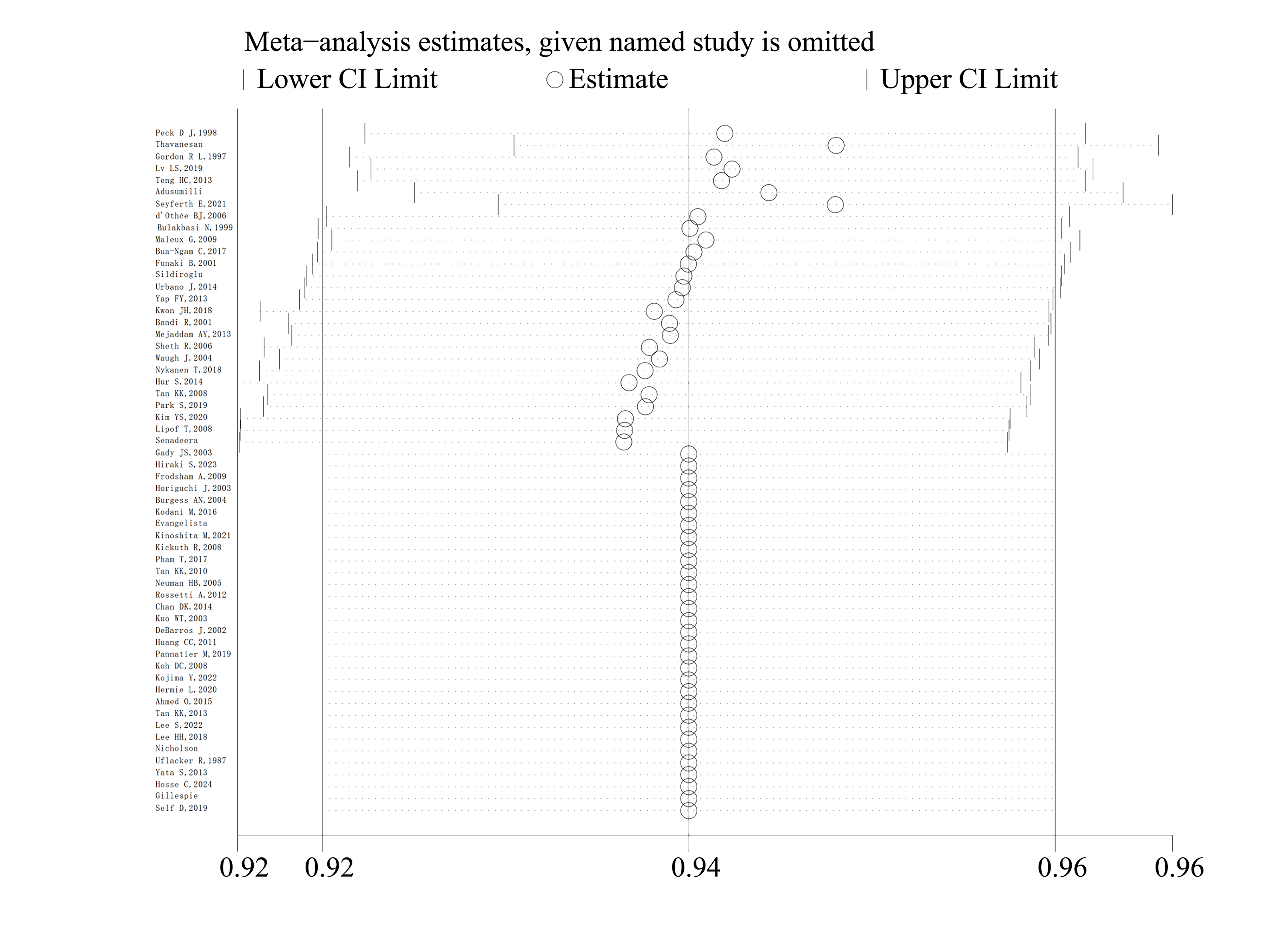


Supplementary Fig 16. Sensitivity analysis of technical success.


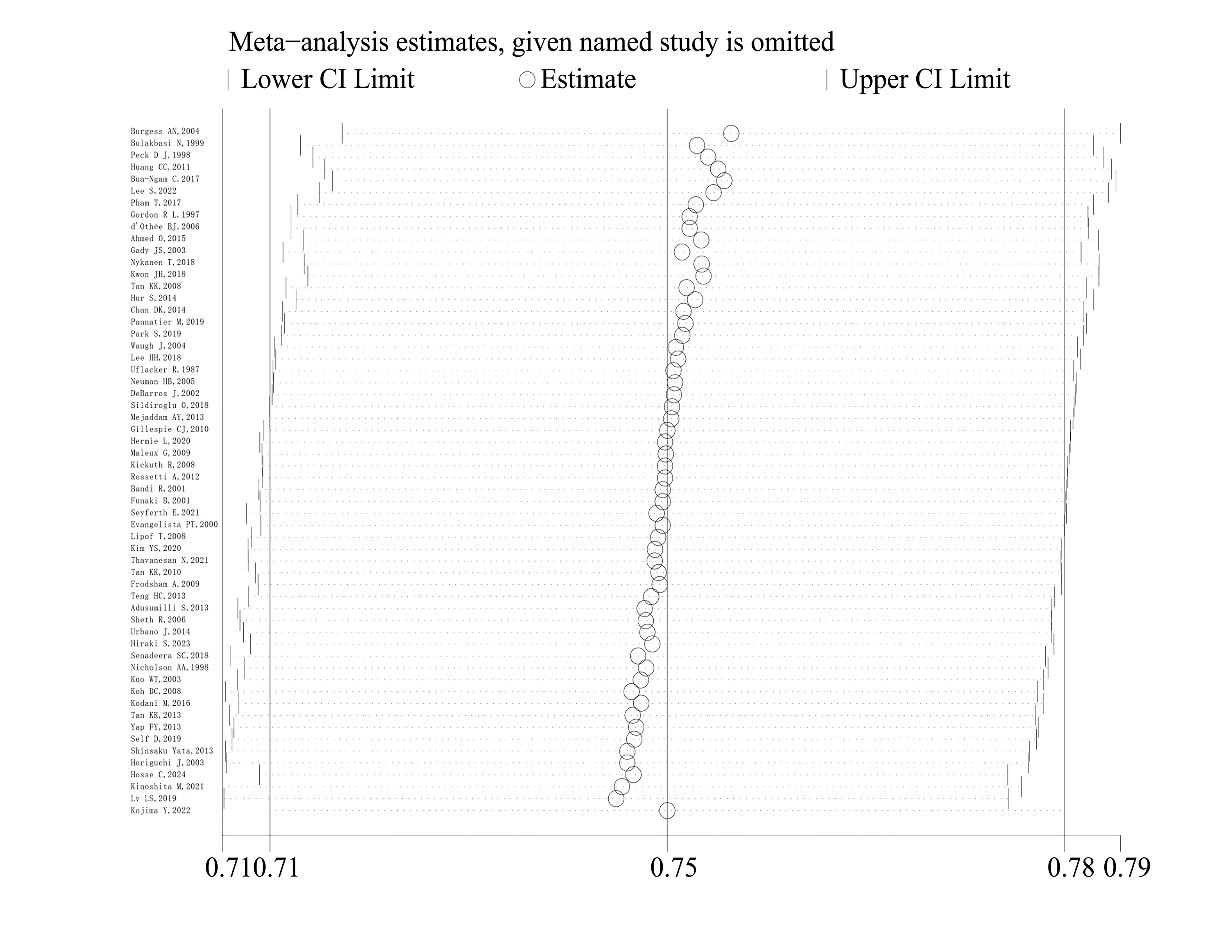


Supplementary Fig 17. Sensitivity analysis of clinical success.


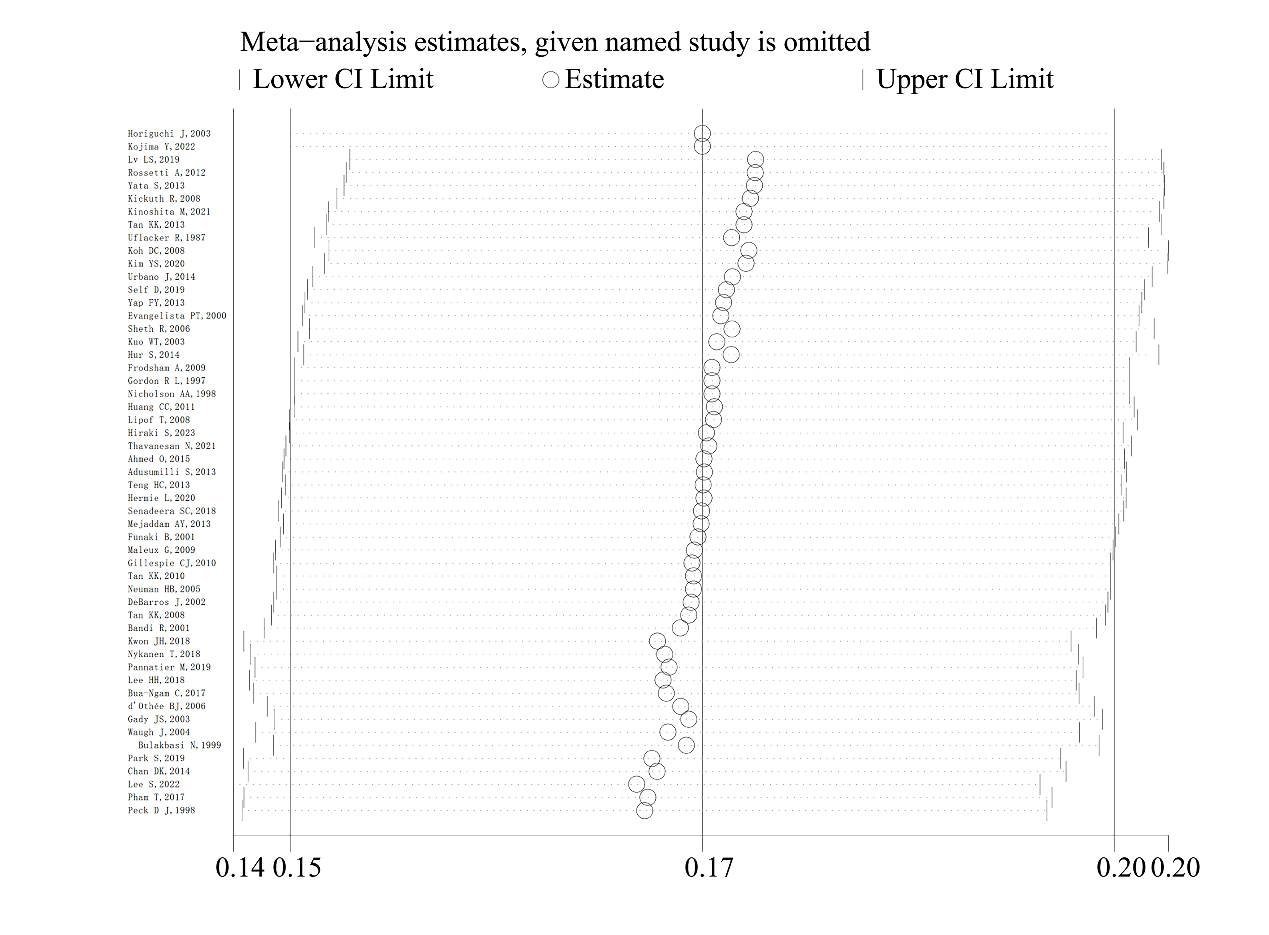


Supplementary Fig 18. Sensitivity analysis of re-bleeding.


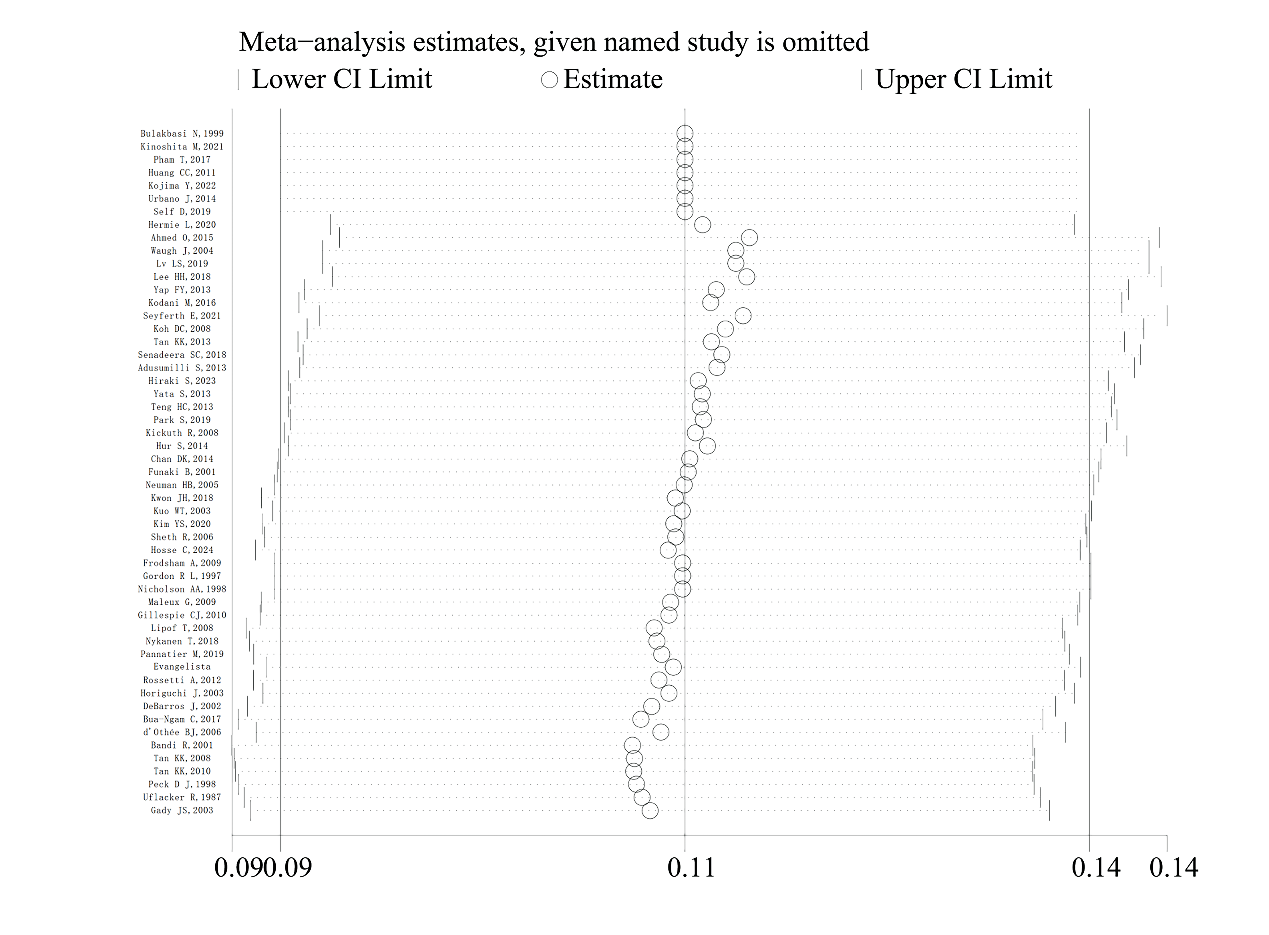


Supplementary Fig 19. Sensitivity analysis of secondary surgery.


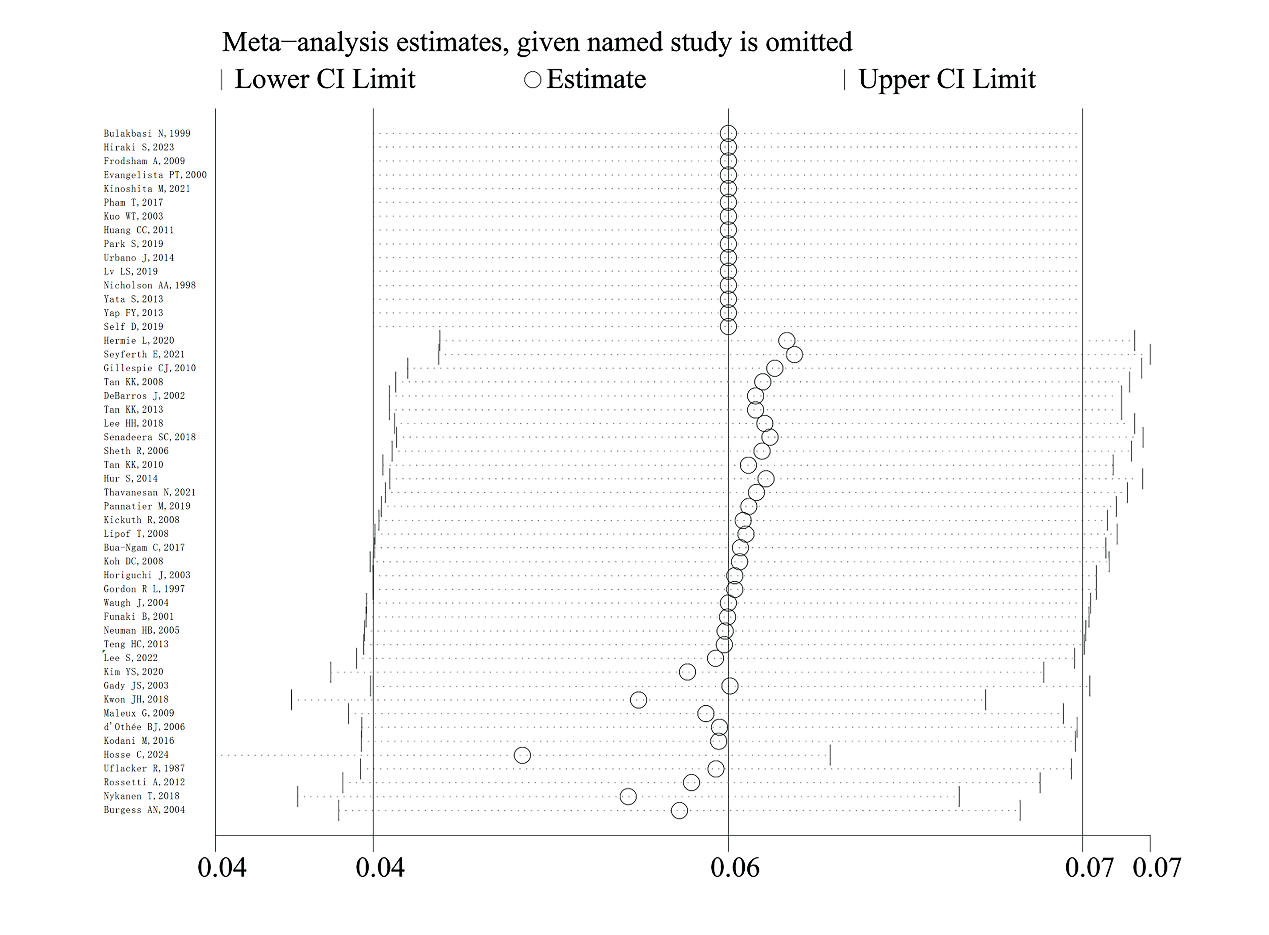


Supplementary Fig 20. Sensitivity analysis of major complications.


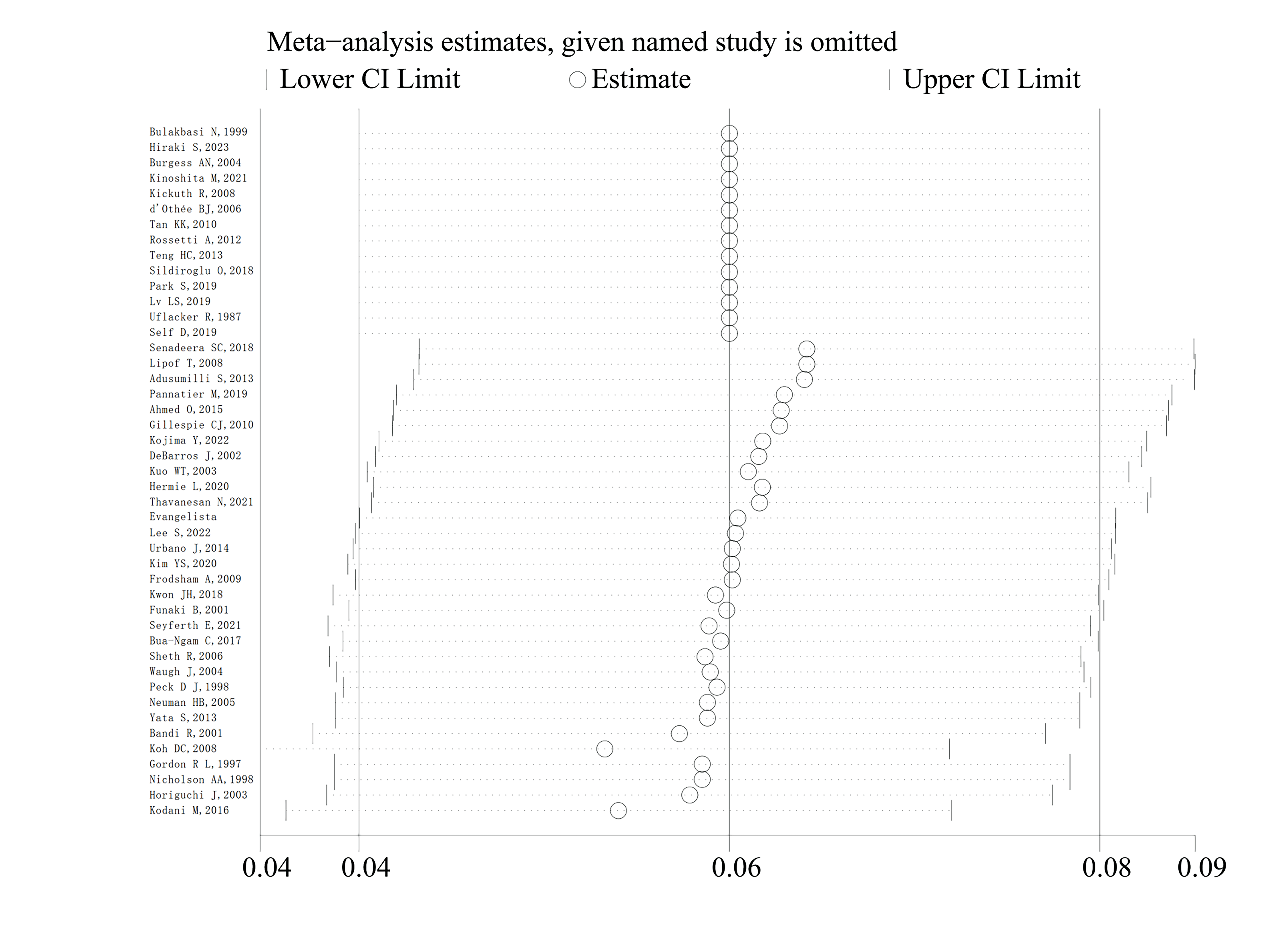


Supplementary Fig 21. Sensitivity analysis of mild ischemia.


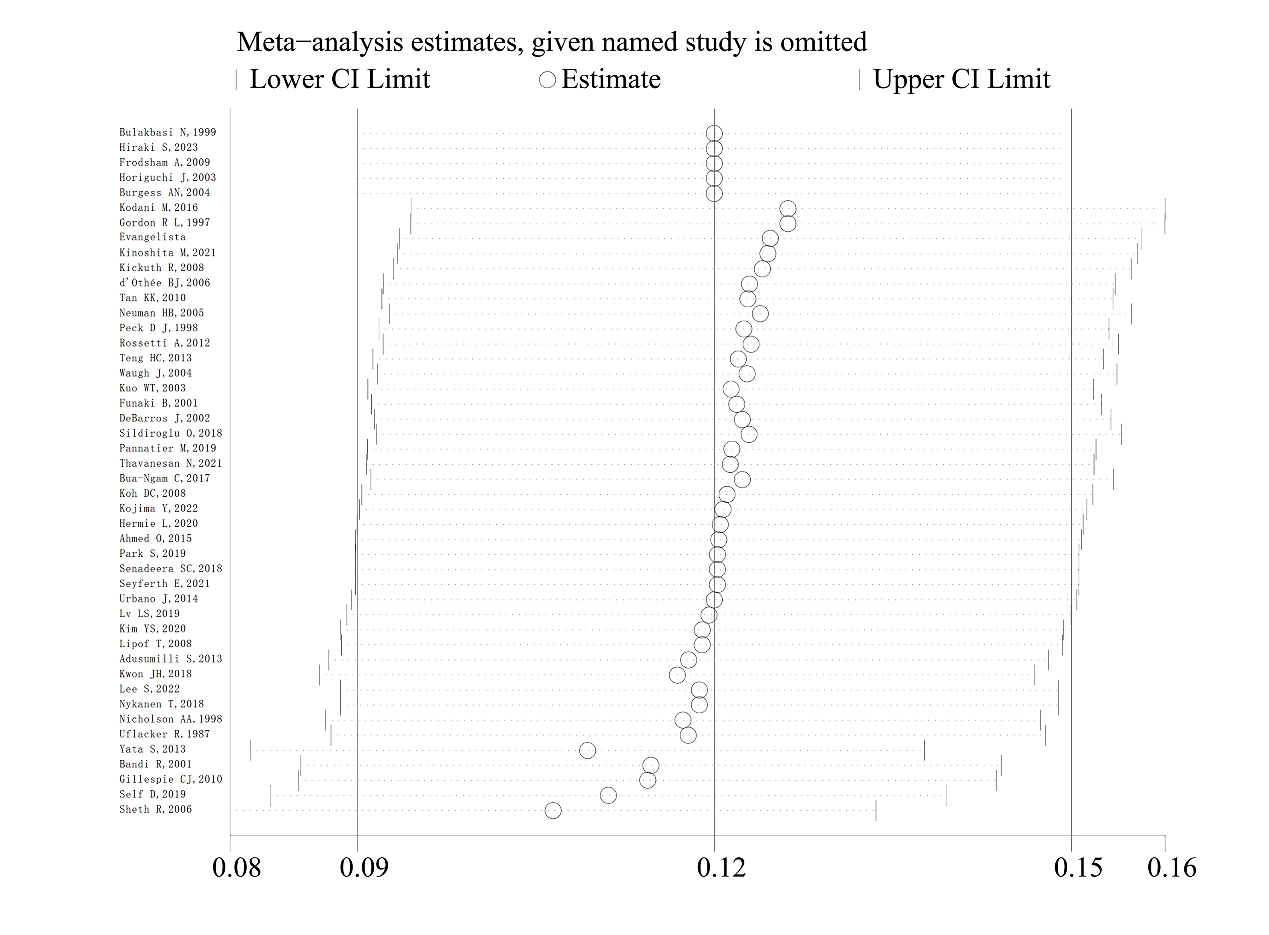


Supplementary Fig 22. Sensitivity analysis of secondary complications.

**Supplementary Table 1. Clinical information and patient characteristics from the eligible studies.**

| **Study** | **Year** | **Design** | **Region** | **Number** | **Materials** | **Technical success rate** | **Clinical success rate** | **Secondary surgery rate** | **Rebleeding intervention rate** | **Major complication** | **Secondary complication** | **First-line treatment** |
| --- | --- | --- | --- | --- | --- | --- | --- | --- | --- | --- | --- | --- |
| R. UFLACK | 1987 | Retrospective | Brazil | 13 | Gelfoam | 13/13 | 9/13 | 5/13 | Death：1 | Infarction 2 | Pain/fever 5 | NR |
| Roy L. Gordon | 1997 | Retrospective | USA | 17 | PVA | 14/17 | 8/14 | 2/14 | TAE1；  Surgery: 1 | Ulcerative colitis 1 | Mild pain 1  Pain/fever 2 | NR |
| David J. Peck | 1998 | Retrospective | Britain | 21 | Gelfoam 、Coil | 17/21 | 8/17 | 6/17 | Surgery：6  Endoscopy：1  Conservative：1  Death：1 | / | Mild ischemia 2 | NR |
| A A Nicholson | 1998 | Retrospective | Britain | 14/38 | Coil | 14/14 | 12/14 | 2/14 | Surgery：2 | 0 | Mild ischemia 3 | Yes |
| Nail Bulakba i | 1999 | Retrospective | Turkey | 10 | PVA、Coil | 9/10 | 4/10 | 0 | TAE：3 | 0 | Pain 7，Fever 5 | Yes |
| Peter T. Evangelista | 2000 | Retrospective | USA | 17 | Coil、PVA、Gelfoam | 17/17 | 13/17 | 3/17 | TAE：1  Surgery：1 | 0 | Mild ischemia 1  Pain 1 | NR |
| Brian Funaki | 2001 | Retrospective | USA | 27 | Coil | 25/27 | 19/25 | 3/25 | Surgery：2  Endoscopy：1  Conservative：2 | Severe ischemia 1  Infarction 1 | Mild ischemia 2 | Yes |
| Bandi R | 2001 | Retrospective | USA | 39 | Coil、PVA、Gelfoam | 37/39 | 28/37 | 9/37 | Surgery：9 | / | Mild ischemia 6 | No |
| John DeBarros | 2002 | Retrospective | USA | 27 | Coil、PVA | 27/27 | 19/27 | 6/27 | TAE：1  Surgery：5 | Severe ischemia 1 | Mild ischemia 1  Pain/fever 2 | No |
| Capt Joshua S. Gady | 2003 | Retrospective | USA | 10 | NR | 10/10 | 6/10 | 4/10 | Surgery：3 | Severe ischemia 1 | NR | NR |
| J. HORIGUCHI | 2003 | Retrospective | Japan | 14 | Gelfoam，Coil | 14/14 | 13/14 | 3/14 | 0 | Muscle fibrosis 1 | Mild ischemia 4  Mild Pain 10  Severe 1 | NR |
| William T. Kuo | 2003 | Retrospective | USA | 22 | Coil、PVA、Gelfoam | 22/22 | 19/22 | 3/22 | Endoscopy：3 | 0 | Mild ischemia 1 | No |
| ADELE N. BURGESS | 2004 | Retrospective | Australia | 15 | Coil、PVA、Gelfoam | 15/15 | 5/15 | NR | NR | Ischemia 4 | Pain 8 | No |
| J Waugh | 2004 | Retrospective | Australia | 27 | Coil、PVA、Gelfoam | 26/27 | 18/26 | 1/26 | TAE：6  Surgery：1  Conservative：1 | Ischemia 2 | Mild ischemia 3 | Yes |
| HEATHER B. NEUMAN | 2005 | Retrospective | USA | 23 | PVA、Coil | 23/23 | 16/23 | 3/23 | TAE：1  Surgery：2  Conservative：2 | Enterostenosis 2 | Mild ischemia 3  Acute renal insufficiency 1  Pseudoaneurysm2 | Yes |
| Bertrand Janne d Othe´ e | 2006 | Retrospective | USA | 19 | Coil | 17/19 | 10/17 | 4/17 | TAE：1  Surgery：2  Conservative：2 | Ischemia 2 | Transient creatinine elevation 1  The coil was transferred into the tibial artery 1 | NR |
| Sheth R | 2006 | Retrospective | India | 52 | Coil、PVA | 50/52 | 41/50 | 7/50 | Surgery：6 | Ischemia 2 | Mild ischemia 5 | No |
| Ralph Kickuth | 2008 | Retrospective | Switzerland | 20 | Coil、PVA | 20/20 | 15/20 | 2/20 | Surgery：1 | Severe ischemia 1 | Inguinal hematoma1 | No |
| D. C. Koh | 2008 | Retrospective | USA | 68 | Coil 、PVA | 68/68 | 59/68 | 5/68 | TAE：3  Surgery：3 | Severe ischemia 3  Infarction 1 | Pain、Fever14 | No |
| Ker-Kan Tan | 2008 | Retrospective | Singapore | 32 | Coil、PVA、Gelfoam | 31/32 | 20/31 | 8/31 | TAE：1  Surgery：4  Endoscopy：1  Death：1 | Severe ischemia 1 | NR | NR |
| T. Lipof | 2008 | Retrospective | USA | 75 | Coil、PVA、Gelfoam | 73/75 | 56/73 | 12/73 | TAE：4  Surgery：8 | Ischemia 4 | Mild ischemia 1 | No |
| Aaron Frodsham | 2009 | Retrospective | USA | 14 | NBCA | 14/14 | 11/14 | 2/14 | Surgery：2 | 0 | Mild ischemia 1 | No |
| Geert Maleux | 2009 | Retrospective | Belgium | 122 | Coil、PVA、Gelfoam、Iodized oil mixture | 39/43 | 29/39 | 6/39 | TAE：2  Surgery：3  Death：3 | Ischemia 4 | NR | No |
| Ker-Kan Tan | 2010 | Retrospective | Singapore | 23 | Coil、PVA | 23/23 | 18/23 | 7/23 | Surgery：5 | Severe ischemia 1 | Hematoma in the groin 1 | No |
| Gillespie CJ | 2010 | Retrospective | Australia | 38 | / | 38/38 | 28/38 | 6/38 | TAE：3  Surgery：5 | Ischemia 1 | Mild ischemia 1 | No |
| Chun-Chieh Huang | 2011 | Retrospective | Taiwan | 27 | NBCA | 27/27 | 13/27 | 0 | TAE：3  Death：1 | 0 | NR | NR |
| Andrea Rossetti | 2012 | Retrospective | Switzerland | 24 | Coil、PVA、Gelfoam | 24/24 | 18/24 | 5/24 | Surgery：1 | Ischemia 4 | Hematoma in the groin 1  Acute renal insufficiency 1 | NR |
| Hui-Chung Teng | 2013 | Retrospective | Taiwan | 26 | Coil | 22/26 | 18/22 | 2/22 | Surgery：4  Death：2 | Ischemia 2 | 0 | NR |
| S. Adusumilli | 2013 | Retrospective | Australia | 71 | Coil、Gelfoam | 61/71 | 50/61 | 5/61 | TAE：3  Surgery：5  Conservative：3 | NR | Colonic mucosa ischemia in 1; Deterioration of renal function 8, inguinal hematoma6, hypersensitivity to contrast media 5, aortic dissection2, inguinal infection 2, leg ischemia 1 | No |
| Ker-Kan Tan | 2013 | Retrospective | Singapore | 27 | Coil、PVA、Gelfoam | 27/27 | 24/27 | 2/27 | TAE：2 | Intestinal obstruction1 | NR | NR |
| Yata S | 2013 | Retrospective | Japan | 23 | NBCA、Coil Gelfoam | 23/23 | 21/23 | 2/23 | TAE：1 | 0 | Mild ischemia 3 Acute ischemia of lower limb 1 | No |
| Mejaddam AY | 2013 | Retrospective | USA | 22 | Coil、PVA | 21/22 | 15/21 | / | 4/21（Unclear） | / | / | No |
| Yap FY | 2013 | Retrospective | USA | 19 | Coil、PVA、Gelfoam | 18/19 | 16/18 | 1/18 | TAE：1  Surgery：1 | 0 | / | No |
| Dedrick Kok Hong Chan | 2014 | Retrospective | Singapore | 26 | Gelfoam、Coil | 26/26 | 17/26 | 3/26 | TAE：1  Surgery：3  Conservative：5 | / | / | No |
| Saebeom Hur | 2014 | Retrospective | Korea | 112 | NBCA，Coil、PVA、Gelfoa，Blood clot | 108/112 | 70/108 | 11/108 | TAE：9  Surgery：2  Endoscopy：1  Conservative：1 | Ischemia 5 | NR | No |
| José Urbano | 2014 | Retrospective | Spain | 31 | Ethylene-vinyl alcohol copolymer | 29/31 | 24/29 | 0 | 3（Unclear） | 0 | Mild ischemia 2 | No |
| O AHMED | 2015 | Retrospective | USA | 40 | Coil | 39/39 | 23/39 | 1/39 | 7（Unclear） | / | Short segmental artery dissection1  Mild ischemia 1 | NR |
| Mika Kodani | 2016 | Retrospective | Japan | 16 | NBCA | 16/16 | 14/16 | 1/16 | / | Intestinal tuberculosis intestinal perforation 1 sigmoid stenosis 1 | Mild ischemia 8 | NR |
| Toan Pham | 2017 | Retrospective | Australia | 18 | Coil、Gelfoam | 18/18 | 10/18 | 0 | TAE：1  Conservative：7 | 0 | NR | NR |
| C.Bua-ngam | 2017 | Retrospective | Thailand | 38 | PVA、Coil、Gelfoam | 35/38 | 17/35 | 8/35 | TAE：1  Surgery：6  Conservative：3 | Severe ischemia 2 | Mild ischemia 3 artery dissection 2 | No |
| Onur Sıldıroğlu | 2018 | Retrospective | USA | 30 | PVA、Coil、Gelfoam | 28/30 | 20/28 | / | / | NR | Inguinal hematoma 1, left colic artery dissection 1, coil straying into jejunal artery 1 | No |
| Sajith C. Senadeera | 2018 | Retrospective | New Zealand | 77 | Coil | 75/77 | 63/75 | 6/75 | TAE：1  Surgery：4  Conservative：9 | Ischemia3 | Mild ischemia 1 | NR |
| Joon Ho Kwon | 2018 | Retrospective | Korea | 134 | NBCA、Coil、Gelfoam | 127/134 | 80/127 | 17/127 | TAE：6  Surgery：7  Endoscopy：10  Conservative：8 | 13/127 | Mild ischemia 10 | No |
| Taina Nykänen | 2018 | Retrospective | Finland | 121 | PVA、Coil、Gelfoam | 53/55 | 32/53 | 9/53 | 14(Unclear) | Severe ischemia 9 | Minor complication 10 | No |
| Han Hee Lee | 2018 | Retrospective | Korea | 52 | NBCA、PVA、Coil、Gelfoam Superfluid fatty iodol | 52/52 | 36/52 | 2/52 | TAE：3  Surgery：2  Endoscopy：2  Conservative：7 | Intestinal infarction 2 | NR | No |
| Megan Pannatier | 2019 | Retrospective | Switzerland | 41 | Coil、NBCA、Iodized oil mixture | 41/41 | 27/41 | 7/41 | TAE：3  Surgery：5  Endoscopy：1  Conservative：2 | Intestinal infarction 2 | Ischemic colitis 1 | NR |
| Suyoung Park | 2019 | Retrospective | Korea | 34 | Coil、Gelfoam NBCA | 33/34 | 22/33 | 3/33 | TAE：3  Surgery：3  Endoscopy：2  Death：3 | 0 | Right common femoral artery thrombosis 1 | No |
| Liang-Shan Lv | 2019 | Retrospective | China | 31 | Coil、Gelfoam PVA | 26/31 | 25/26 | 1/26 | Surgery：1 | 0 | 0 | No |
| Self D | 2019 | Retrospective | Australia | 19 | Coil | 19/19 | 17/19 | 0 | TAE：2 | 0 | 0 | No |
| Laurens Hermie | 2020 | Retrospective | Belgium | 66 | NBCA、Coil PVA | 66/66 | 49/66 | 1/66 | 12（Unclear） | Intestinal infarction 1 | Mild ischemia 3  Acute renal failure 2 | NR |
| Yong Seek Kim | 2020 | Retrospective | Korea | 74 | NBCA、Coil、Gelfoam | 72/74 | 56/72 | 10/72 | TAE：2  Surgery：3  Endoscopy：2 | Intestinal infarction 7 | Mild ischemia 5 | No |
| MitsuhiroKinoshita | 2021 | Retrospective | Japan | 17 | Coil | 17/17 | 16/17 | 0 | Conservative：1 | 0 | 0 | No |
| Navamayooran Thavanesan | 2021 | Retrospective | New Zealand | 79 | Coil、Gelfoam | 64/79 | 50/64 | / | 11（Unclear） | Ischemia 3 | Vascular injury 2  Contrast nephropathy1 | NR |
| Elisabeth Seyferth | 2021 | Retrospective | USA | 134 | PVA、Coil、Gelfoam | 134/154 | 102/134 | 9/13 | / | Severe ischemia 3 | Mild ischemia 11  Thrombosis at the approach site1 | No |
| Yuki Kojima | 2022 | Retrospective | Japan | 39 | Coil | 29/29 | 29/29 | 0 | 0 | / | ischemic ulcer1  Fever 1 Pain1 | Yes |
| Shinhaeng Lee | 2022 | Retrospective | Korea | 31 | NR | 31/31 | 16/31 | NR | 12（Unclear） | Intestinal infarction 3 | Mild ischemia 2  Arterial dissection2 | No |
| Sakiko Hiraki | 2023 | Retrospective | Japan | 12 | IPM/CS mixture | 12/12 | 10/12 | 1/12 | Surgery：1  Endoscopy：1 | 0 | 0 | Yes |
| Hosse C | 2024 | Retrospective | Germany | 128 | NBCA、PVA、Coil | 128/128 | 120/128 | 18/128 | / | 18/128 | / | No |

**Supplementary Table 2 Quality assessment of included studies.**

| **A. The JBl Critical Appraisal Checklist for Case Series for included retrospective single-arm studies** | | | | | | | | | | | |
| --- | --- | --- | --- | --- | --- | --- | --- | --- | --- | --- | --- |
| **Study** | **Q1** | **Q2** | **Q3** | **Q4** | **Q5** | **Q6** | **Q7** | **Q8** | **Q9** | **Q10** | **Overall appraisal** |
| Uflacker R,1987 | yes | yes | yes | yes | yes | yes | yes | yes | yes | yes | Include |
| Gordon R L,1997 | yes | yes | yes | yes | yes | yes | yes | yes | yes | yes | Include |
| Peck D J,1998 | yes | yes | yes | yes | yes | yes | yes | yes | yes | yes | Include |
| Nicholson AA,1998 | yes | yes | yes | no | yes | yes | yes | yes | yes | yes | Include |
| Bulakbasi N,1999 | yes | yes | yes | yes | yes | yes | yes | yes | yes | yes | Include |
| Evangelista PT,2000 | yes | yes | yes | yes | yes | yes | yes | yes | yes | yes | Include |
| Funaki B,2001 | yes | yes | yes | yes | yes | yes | yes | yes | yes | yes | Include |
| Bandi R,2001 | yes | yes | yes | no | yes | yes | yes | yes | yes | yes | Include |
| DeBarros J,2002 | yes | yes | yes | yes | yes | yes | yes | yes | yes | yes | Include |
| Gady JS,2003 | yes | yes | yes | yes | yes | yes | yes | yes | yes | yes | Include |
| Horiguchi J,2003 | yes | yes | yes | yes | yes | yes | yes | yes | yes | yes | Include |
| Kuo WT,2003 | yes | yes | yes | yes | yes | yes | yes | yes | yes | yes | Include |
| Burgess AN,2004 | yes | yes | yes | no | yes | yes | yes | yes | yes | yes | Include |
| Waugh J,2004 | yes | yes | yes | yes | yes | yes | yes | yes | yes | yes | Include |
| Neuman HB,2005 | yes | yes | yes | yes | yes | yes | yes | yes | yes | yes | Include |
| d'Othée BJ,2006 | yes | yes | yes | yes | yes | yes | yes | unclear | yes | yes | Include |
| Sheth R,2006 | yes | yes | yes | no | yes | yes | yes | yes | yes | yes | Include |
| Kickuth R,2008 | yes | yes | yes | yes | yes | yes | yes | yes | yes | yes | Include |
| Koh DC,2008 | yes | yes | yes | yes | yes | yes | yes | yes | yes | yes | Include |
| Tan KK,2008 | yes | yes | yes | yes | yes | yes | yes | yes | yes | yes | Include |
| Lipof T,2008 | yes | yes | yes | yes | yes | yes | yes | yes | yes | yes | Include |
| Frodsham A,2009 | yes | yes | yes | yes | yes | yes | yes | yes | yes | yes | Include |
| Maleux G,2009 | yes | yes | yes | yes | yes | yes | yes | yes | yes | yes | Include |
| Tan KK,2010 | yes | yes | yes | yes | yes | yes | yes | yes | yes | yes | Include |
| Gillespie CJ,2010 | yes | yes | yes | no | yes | yes | yes | unclear | yes | yes | Include |
| Huang CC,2011 | yes | yes | yes | yes | yes | yes | yes | yes | yes | yes | Include |
| Rossetti A,2012 | yes | yes | yes | yes | yes | yes | yes | yes | yes | yes | Include |
| Teng HC,2013 | yes | yes | yes | yes | yes | yes | yes | yes | yes | yes | Include |
| Adusumilli S,2013 | yes | yes | yes | yes | yes | yes | yes | yes | yes | yes | Include |
| Tan KK,2013 | yes | yes | yes | yes | yes | yes | yes | yes | yes | yes | Include |
| Yata S,2013 | yes | yes | yes | yes | yes | yes | yes | yes | yes | yes | Include |
| Mejaddam AY,2013 | yes | yes | yes | no | yes | yes | yes | yes | yes | yes | Include |
| Yap FY,2013 | yes | yes | yes | no | yes | yes | yes | yes | yes | yes | Include |
| Chan DK,2014 | yes | yes | yes | yes | yes | yes | yes | yes | yes | yes | Include |
| Hur S,2014 | yes | yes | yes | yes | yes | yes | yes | yes | yes | yes | Include |
| Urbano J,2014 | yes | yes | yes | yes | yes | yes | yes | yes | yes | yes | Include |
| Ahmed O,2015 | yes | yes | yes | yes | yes | yes | yes | yes | yes | yes | Include |
| Kodani M,2016 | yes | yes | yes | no | yes | yes | yes | yes | yes | yes | Include |
| Pham T,2017 | yes | yes | yes | yes | yes | yes | yes | yes | yes | yes | Include |
| Bua-Ngam C,2017 | yes | yes | yes | no | yes | yes | yes | yes | yes | yes | Include |
| Sildiroglu O,2018 | yes | yes | yes | no | yes | yes | yes | unclear | yes | yes | Include |
| Senadeera SC,2018 | yes | yes | yes | yes | yes | yes | yes | yes | yes | yes | Include |
| Kwon JH,2018 | yes | yes | yes | yes | yes | yes | yes | yes | yes | yes | Include |
| Nykanen T,2018 | yes | yes | yes | yes | yes | yes | yes | yes | yes | yes | Include |
| Lee HH,2018 | yes | yes | yes | yes | yes | yes | yes | yes | yes | yes | Include |
| Park S,2019 | yes | yes | yes | yes | yes | yes | yes | yes | yes | yes | Include |
| Lv LS,2019 | yes | yes | yes | no | yes | yes | yes | yes | yes | yes | Include |
| Self D,2019 | yes | yes | yes | no | yes | yes | yes | yes | yes | yes | Include |
| Hermie L,2020 | yes | yes | yes | yes | yes | yes | yes | yes | yes | yes | Include |
| Kim YS,2020 | yes | yes | yes | yes | yes | yes | yes | yes | yes | yes | Include |
| Kinoshita M,2021 | yes | yes | yes | yes | yes | yes | yes | yes | unclear | yes | Include |
| Thavanesan N,2021 | yes | yes | yes | yes | yes | yes | yes | yes | yes | yes | Include |
| Seyferth E,2021 | yes | yes | yes | yes | yes | yes | yes | yes | unclear | yes | Include |
| Lee S,2022 | yes | yes | yes | yes | yes | yes | yes | yes | yes | yes | Include |
| Hiraki S,2023 | yes | yes | yes | yes | yes | yes | yes | yes | yes | yes | Include |
| Hosse C,2024 | yes | yes | yes | no | yes | yes | yes | yes | yes | yes | Include |

**B. The Newcastle–Ottawa Scale was used to assess the other suitable studies**

| **Study** | **Selection** | **Comparability** | **Outcome** | **Quality score** |
| --- | --- | --- | --- | --- |
| Pannatier M,2019 | 4 | 2 | 3 | 9 |
| Kojima Y,2022 | 4 | 2 | 2 | 8 |
